# Morphometric network-based abnormalities correlate with psychiatric comorbidities and gene expression in *PCDH19-*related developmental and epileptic encephalopathy

**DOI:** 10.1038/s41398-024-02753-x

**Published:** 2024-01-18

**Authors:** Matteo Lenge, Simona Balestrini, Antonio Napolitano, Davide Mei, Valerio Conti, Giulia Baldassarri, Marina Trivisano, Simona Pellacani, Letizia Macconi, Daniela Longo, Maria Camilla Rossi Espagnet, Simona Cappelletti, PCDH19 Clinical Study Group, Annarita Ferrari, Tiziana Pisano, Federico Sicca, Pasquale Striano, Ludovico D’Incerti, Carmen Barba, Nicola Specchio, Renzo Guerrini

**Affiliations:** 1 Child Neurology Unit and Laboratories, Neuroscience Department, Meyer Children’s Hospital IRCCS, 50139 Florence, Italy; 2 Medical Physics Department, Bambino Gesù Children’s Hospital, IRCCS, 00100 Rome, Italy; 3 Neurology, Epilepsy and Movement Disorders, Bambino Gesù Children’s Hospital, IRCCS, Full Member of European Reference Network EpiCARE, 00165 Rome, Italy; 4 Pediatric Radiology Unit, Meyer Children’s Hospital IRCCS, 50139 Florence, Italy; 5 Functional and Interventional Neuroimaging Unit, Bambino Gesù Children’s Hospital, IRCCS, 00165 Rome, Italy; 6 IRCCS Stella Maris Foundation, Pisa, Italy; 7 Neuroscience Department, Meyer Children’s Hospital IRCCS, Florence, Italy; 8 Paediatric Neurology and Muscular Diseases Unit, IRCCS ‘G. Gaslini’ Institute, Genova, Italy

**Keywords:** Autism spectrum disorders, Diagnostic markers

## Abstract

Protocadherin-19 (*PCDH19*) developmental and epileptic encephalopathy causes an early-onset epilepsy syndrome with limbic seizures, typically occurring in clusters and variably associated with intellectual disability and a range of psychiatric disorders including hyperactive, obsessive-compulsive and autistic features. Previous quantitative neuroimaging studies revealed abnormal cortical areas in the limbic formation (parahippocampal and fusiform gyri) and underlying white-matter fibers. In this study, we adopted morphometric, network-based and multivariate statistical methods to examine the cortex and substructure of the hippocampus and amygdala in a cohort of 20 *PCDH19*-mutated patients and evaluated the relation between structural patterns and clinical variables at individual level. We also correlated morphometric alterations with known patterns of PCDH19 expression levels. We found patients to exhibit high-significant reductions of cortical surface area at a whole-brain level (left/right *p*_*value*_ = 0.045/0.084), and particularly in the regions of the limbic network (left/right parahippocampal gyri *p*_*value*_ = 0.230/0.016; left/right entorhinal gyri *p*_*value*_ = 0.002/0.327), and bilateral atrophy of several subunits of the amygdala and hippocampus, particularly in the CA regions (head of the left CA3 *p*_*value*_ = 0.002; body of the right CA3 *p*_*value*_ = 0.004), and differences in the shape of hippocampal structures. More severe psychiatric comorbidities correlated with more significant altered patterns, with the entorhinal gyrus (*p*_*value*_ = 0.013) and body of hippocampus (*p*_*value*_ = 0.048) being more severely affected. Morphometric alterations correlated significantly with the known expression patterns of PCDH19 (*r*_*value*_ = -0.26, *p*_*spin*_ = 0.092). *PCDH19* encephalopathy represents a model of genetically determined neural network based neuropsychiatric disease in which quantitative MRI-based findings correlate with the severity of clinical manifestations and had have a potential predictive value if analyzed early.

## Introduction

Protocadherin-19 (*PCDH19*) gene mutations cause an infantile-onset epilepsy syndrome with limbic seizures, typically occurring in clusters and variably associated with intellectual disability and a range of psychiatric disorders including autistic features [1]. Cognitive and affective dysfunctions in *PCDH19* developmental and epileptic encephalopathy (DEE) impose a major medical and socio-economic burden and are increasingly recognized as core disease dimensions that can affect patients’ quality of life even more than the seizures themselves [2]. Kolc and colleagues [3] reviewed 271 PCDH19-variant individuals and found hyperactive, autistic and obsessive-compulsive features to be the most frequently observed neuropsychiatric manifestations, while no genotype-phenotype associations emerged in individuals with recurrent variants, or in the group overall. An earlier seizure onset was significantly associated with more severe intellectual disability, but no correlations were observed between the type or location of *PCDH19* mutation, neuropsychiatric profile, and age at seizure onset.

PCDH19 *is* a calcium dependent cell-adhesion molecule involved in neuronal circuit formation. In individuals with *PCDH19*-gene mutations, gross human brain morphology appears usually normal on MRI [4], with mild, ill-defined malformations of the cortical mantle having been described in isolated patients [5]. However, quantitative neuroimaging reveals abnormal cortical areas in the limbic formation and underlying white-matter fibers [6]. In patients with earlier onset of seizures, worse autistic manifestations, and cognitive impairment, structural abnormalities in the limbic areas are more pronounced [6]. Using MRI, a correlation between abnormal mesio-temporal anatomy and autism has been reported in different etiological settings [7–10]. MRI has further disclosed altered patterns of brain expansion in children with autism [11], but a correlation between specific mutations and autism remains to be clarified in *PCDH19*-related phenotypes [3].

Developmental abnormalities involving the limbic structures, particularly the parahippocampal and entorhinal gyri, likely represent a measurable anatomic consequence of the altered expression of the PCDH19 protein on cortical folding and white matter organization in these anatomic areas [12], and are functionally reflected in the phenotypic features involving neuropsychiatric symptoms, cognitive and communicative skills and local epileptogenesis [3]. Recent studies on animal models have contributed to clarify how *PCDH19* mutations alter brain network formation and maintenance [13], and have identified mechanisms by which focal mosaic mutations disrupt cortical networks [14] and impair neuronal activity and connectivity [15]. Exploring the structural organization of the limbic regions in *PCDH19*-related phenotypes could benefit from adopting network-based algorithms [16]. Such approaches, most often used to study atrophic patterns, may unveil broader inter-associations between brain regions, contributing to a deeper understanding of the underlying developmental relationships at a whole-brain level. Cortico-subcortical network alterations in epilepsy have been associated with genetic risk factors at large [17], but the links between neurobiological mechanisms and cortical features in single-gene developmental and epileptic encephalopathies remain poorly explored. Open databases focused on transcriptomic and proteomic provide a great resource for integrating macroscale neuroimaging observations with molecular-level spatial variances [18]. Syndrome-specific morphometric alterations may indeed reflect the spatial expression patters of *PCDH19* and potentially give insight into the intricate interplay between neuroimaging data and molecular-scale information.

Neuroimaging research has moved on to disentangle the contributions of different hippocampal subregions and adjacent cortices, so as to bridge the gap between rodent and human data [19]. In previous quantitative MRI study of *PCDH19*-related encephalopathy [6], no volumetric alterations emerged in the hippocampus at a whole level, although surface patterns in the mesio-temporal cortex were abnormal. However, the hippocampus has a key role in limbic epilepsies [20], and PCDH19 has a prevalent expression in the hippocampal structures [21], particularly in the *Cornu Ammonis* (CA) [22]. Latest studies on murine models have highlighted that PCDH19 impairment contributes to develop structural and functional synaptic defects in the hippocampus [15].

In this study, we adopted morphometry and network-based models to detect abnormal structural patterns of brain regions in patients with *PCDH19*-related epilepsy, previously analyzed in the cortex and white matter, and comparatively studied patients’ subgroups stratified by different clinical severity at the individual level. We also adopted advanced neuroimaging methods to further investigate the substructure and shape of the hippocampus, CA and amygdala subunits, and evaluated the relation between structural patterns of abnormality and spatial expression of PCDH19. We found the limbic circuit to represent a structural epicenter of the anatomic alteration of the cortex and several hippocampal substructures to exhibit reduced volumes. We could also demonstrate that patients exhibiting significantly more severe atrophic patterns experienced more severe psychiatric comorbidities.

## Patients and Methods

### Participants

We studied a cohort of 20 patients seen at two tertiary pediatric neurology centers (Meyer Children’s Hospital IRCCS, 11 patients and 22 controls; IRCCS Bambino Gesù Pediatric Hospital, 9 patients and 18 controls). Patients (mean age 14.1 years, median 13.6, range 2.3-26.6) had been included in previous publications [1, 4, 6, 23, 24] and carried pathogenic *PCDH19* gene mutations (canonical transcript NM_001184880.1) identified by next-generation sequencing, Sanger sequencing or multiplex ligation-dependent probe amplification. To mitigate confounding effects, we enrolled 40 controls that were matched with patients for acquisition center, sex and age ( ± 1year, mean age 14.2 years, median 13.4, range 3.4–27.0) and were healthy individuals who had received an MRI for uncomplicated headache.

#### Clinical and genetic data

For each patient, we collected clinical and genetic data using the REDCap (Research Electronic Data Capture) [25] tool hosted at Meyer Children’s Hospital IRCCS. Clinical data considered for the purpose of this study included age at seizure onset, seizure type, ictal and interictal EEG characteristics, neuropsychology testing and psychiatric manifestations (Table 1). At seizure onset (mean 10.8 months, median 8.0, range 4–28), 18 patients (90%) manifested focal seizures, two had focal to bilateral tonic and tonic-clonic seizures, while at follow-up 16 patients (80%) manifested more seizure types, i.e. focal, tonic, tonic-clonic and absences. The triggering factor for seizures was a febrile illness in 80% of patients. Ten patients presented focal seizures with prominent affective symptoms, suggesting an epileptogenic dysfunction involving the limbic system.

**Table 1:** Genetic, MRI, clinical, cognitive and psychiatric data of *PCDH19*-mutation group.

No.	Age at MRI / Sex	*PCDH19* mutation cDNA/ protein	Inheritance	MRI findings	Seizure onset (months)	Seizure type (onset)	Habitual seizures	Treatment	Limbic seizures	Cognitive level	DSM5 classification	Grade of psychiatric disorder
1a	9.4 / F	c.1769T>G, p.Val590Gly	father	None	8	Fo	Yes	VPA, ZNS, LZM, OLA, CLOR	Yes	Moderate ID	ODD, CD, OCD	Severe
2a	9.4 / F	c.1769T>G, p.Val590Gly	father	None	6	Fo	Yes	VPA, ZNS, RIS, AL	Yes	Moderate ID	ODD, CD, OCD	Severe
3	12.0 / F	c.688 G > T, p.Asp230Tyr	de novo	Hyperintensity in body of L hippocampus	16	Fo	Yes	CBZ	No	Normal	None	None
4	13.6 / F	exon 1 + upstream deletion, n.a.	de novo	None	14	Fo	No	VPA, CLB	No	Normal	DD, AD, ADHD, CD	Moderate
5	2.3 / M	c.1020 T > A, p.Asn340Lys mosaic	de novo	None	5	Fo	Yes	PHT, PB, CLB	No	Mild ID	None	None
6	3.4 / F	c.2338 A > T, p.Lys780*	de novo	None	24	Fo	Yes	CBZ	No	Normal	ODD, CD, IED	Severe
7b	18.9 / F	c.242 T > G, p.Leu81Arg	de novo	None	6	Fo	No	LCM, CLB, FC, CLOT	Yes	Severe ID	ASD, CD	Moderate
8b	18.9 / F	c.242 T > G, p.Leu81Arg	de novo	None	6	Fo	Yes	LCM, CLB, FC, CLOT	Yes	Severe ID	ASD, CD	Moderate
9	11.1 / F	c.465 T > A, p.Asp155Glu	de novo	None	4	Fo	Yes	N.A.	N.A.	Severe ID	N.A.	N.A.
10^a^	20.6 / F	c.1300_1301delCA, p.Gln434Glufs*11	de novo	Incomplete inversion of L hippocampus	14	Fo	Yes	VPA, TPM, CLB	Yes	Moderate ID	ODD, OCD	Moderate
11	23.7 / F	c.1019 A > G, p.Asn340Ser	mother	None	6	Fo	Yes	VPA, TPM, DEL	Yes	Mild ID	ODD, CD, AtD	Mild
12	17.8 / F	c.1129 G > C, p.Asp377His	de novo	None	8	Fo	No	STP, CLB, VPA, RIS	No	Moderate ID	ASD	Severe
13	16.8 / F	c.2676-6 A > G, n.a.	de novo	None	7	Fo	Yes	STP, CLB, VPA, RIS	No	Moderate ID	ASD, IED, CD	Severe
14	7.6 / M	c.1352 C > T, p. Pro451Leu mosaic	de novo	None	10	T	No	STP, VAP, CLB	Yes	Normal	SCD, AtD	Moderate
15	22.5 / F	c.1973T>G, p.Val658Gly	mother	Hypotrophy of cerebellar vermis, anterior cerebellum	5	Fo	Yes	STP, VPA, PER, CNZ	No	Mild ID	ASD, IED, CD	Mild
16^a^	44.3 / F	c.706 C > T, p.Pro236Ser	de novo	None	10	Fo	No	None	Yes	Moderate ID	ASD	Mild
17	8.0 / F	c.1098 C > G, p.Tyr366*	de novo	None	11	Fo	No	VPA, LEV	No	Mild ID	ASD	Moderate
18	9.2 / F	c.1178 C > T, p.Pro393Leu	de novo	None	20	Fo	Yes	VPA	No	Mild ID	ASD, AtD	Mild
19	16.2 / F	c.958dupG, p.Asp320Glyfs*22	de novo	None	28	TC	No	LEV, CBZ	Yes	Normal	None	None
20	26.3 / F	c.1298 T > C, p.Leu433Pro	de novo	None	8	Fo	No	VPA, CBZ	Yes	Normal	OCD	Mild

Cognitive level was classified as (0) “normal”, (1) “mild ID”, (2) “moderate ID” or (3) “severe ID”. The severity of psychiatric comorbidities was classified on an empirical basis as (0) “None”, (1) “Mild”, (2) “Moderate” and (3) “Severe”, considering a severity range spanning from the ‘mild’ category (1) where the psychiatric disorder would cause minimum functional impairment and the symptoms would be considered mild, to the ‘severe’ category (3) where there would be significant functional individual and social disruption and the symptoms would be considered severe or very severe. Abbreviations: y years, a 1st twin pair, b 2nd twin pair, *M* male, *F* female, *L* left, *N.A*. not available, *Fo* focal, *T* tonic, *TC* tonic-clonic, *A* absences, *ASM* anti-seizure medications, *AL* alprazolam, *CBZ* carbamazepine, *CLOR* clorpromazina, *CLOT* clotiapina, *CNZ* clonazepam, *DEF* deflazacort, *DEL* delorazepam, *FC* fluoxetina Cloridrato, *LCM* lacosamide, *LEV* levetiracetam, *LZM* lorazepam, *OLA* olanzapina, *PB* phenobarbital, *PER* perampanel, *PHT* phenytoin, *RIS* risperidone, *STP* stiripentol, *TMP* topiramate, *VPA* valproic acid (valproate), *ZNS* zonisamide, *ID* intellectual disability, DSM5 Diagnostic and Statistical Manual of Mental disorders, 5th edition [26], DSM5 classification: *ODD* oppositional defiant disorder, *CD* conduct disorder, *OCD* obsessive compulsive disorder, *DD* depressive disorder, *AD* anxiety disorder, *ADHD* attention deficit and hyperactivity disorder, *IED* intermittent explosive disorder, *ASD* autism spectrum disorder, *AtD* attention deficit; ^a^excluded from the hippocampal analysis.

Formal cognitive assessment was performed according to age and verbal function, using one or more of the following neuropsychological tests: Brunet‐Lezine, Griffiths mental development scale, Bayley Scales of Infant and Toddler Development (Bayley‐III), Leiter International Performance Scale‐Revised (Leiter‐R), Raven’s Coloured Progressive Matrices, Stanford‐Binet test, Wechsler Preschool and Primary Intelligence test (WPPSI), Wechsler Intelligence Scale for Children (WISC), Wechsler Adult Intelligence Scale | Fourth Edition (WAIS‐IV), and Mini‐Mental State Examination (MMSE). Intellectual disability (ID) occurred in 14 patients (70%) (six with moderate level, five with mild, three with severe ID), while in six patients cognitive level was normal. Neurodevelopmental and psychiatric disorders were classified according to the Diagnostic and Statistical Manual of Mental disorders, 5th edition [26]. Most patients (17, 85%) developed psychiatric and behavioral disorders, including conduct disorder (*n* = 10), autism spectrum disorder (*n* = 9), oppositional defiant disorder (*n* = 5), obsessive-compulsive disorder (*n* = 4), intermittent explosive disorder (*n* = 3), attention deficit with or without hyperactivity disorder (*n* = 3), depressive and anxiety disorder (*n* = 1), social communication disorder (*n* = 1). The severity of psychiatric comorbidities was classified on an empirical basis as (0) “None”, (1) “Mild”, (2) “Moderate” and (3) “Severe”, considering a severity range spanning from the ‘mild’ category (1) where the psychiatric disorder would cause minimum functional impairment and the symptoms would be considered mild, to the ‘severe’ category (3) where there would be significant functional individual and social disruption and the symptoms would be considered severe or very severe.

Genetic testing revealed that all patients carried pathogenic *PCDH19* mutations, 14 were missense substitutions, six were truncating (two stopgain, two frame-shift, one genomic deletion and one splicing). Eighteen patients were females, and two were males who carried mosaic *PCDH19* mutations. In sixteen patients (80%) mutations occurred de novo, while in four they had been inherited (two from father, two from mother).

### MRI data acquisition

We performed MRI acquisitions in participants adopting the same protocol on two 3 T systems (Achieva, Philips Healthcare, The Netherlands, at Meyer Hospital IRCCS; Magnetom Skyra, Siemens Healthineers, Germany, at IRCCS Bambino Gesù Hospital). MRI assessment included: 3D T1-weigthed fast-spoiled gradient echo (3D-T1), 3D T2-weighted Fluid Attenuation Inversion Recovery, T2-weighted Fast Spin-Echo, T2*-weighted Gradient-eco, and 2D T1-weigthed acquisitions. We acquired 3D-T1 whole-brain MRI by setting both 3 T scanners with the same sequence parameters (acquisition plane sagittal, TR/TE 8.0/3.7 ms, matrix 240 × 240, flip angle 8°, slice thickness 1 mm/no gap, FOV [240×240] mm^2^, number of slices 191, voxel resolution [1.0 × 1.0 × 1.0] mm^3^, acquisition time 6 min, 32 s). The study was approved by the Pediatric Ethics Committee of the Tuscany Region, Italy, in the context of the DESIRE project (FP7 EU call, grant agreement 602531) and its extension by the DECODE-EE (Tuscany Region Call for Health 2018) and the Human Brain Optical Mapping (Fondazione Cassa di Risparmio di Firenze 2021) projects. For all patients and controls, written informed consent for research use of clinical and genetic data was obtained from patients or their parents, or legal guardians in the case of minors or those with intellectual disability.

### MRI data analysis

#### Visual analysis

Brain MRI images were reviewed by pediatric neuroradiologists looking for structural abnormalities and artifacts that could hamper computational analyses.

#### Morphometric analysis of cortical regions

We processed T1-3D MRI of each subject using the FreeSurfer processing pipeline (version 7.2.0, https://surfer.nmr.mgh.harvard.edu). The processing pipeline implies several stages, including segmentation of subcortical structures [27] segmentation of the cortical regions, and reconstruction of surfaces demarcating the boundaries between white and gray matter (*white surface*) and between gray matter and cerebrospinal fluid (CSF, *pial surface*) [28]. We performed the parcellation of the cortical mantle to divide the cortical surfaces into distinct gyral and sulcal regions [29] and generated detailed 3D high-resolution reconstructions for both *white* and *pial* surfaces for each hemisphere. To ensure accuracy, we carried out visual inspections and manual corrections on the subcortical segmentations and cortical surface reconstructions, when appropriate.

We conducted an analysis of the morphometric characteristics of the parceled regions of interest (ROIs). Our focus was to investigate cortical anatomy as it results from its developmental process and, to achieve this, we measured cortical thickness (CT), surface area (SA) and cortical volume (CV) for every vertex of the cortical surfaces. Cortical thickness, as quantified by measuring the distance between the white and pial surfaces [30], provides insights into neuronal migration and postmigrational organization [31], as well as on gray matter [32]. We determined the SA by summing the areas of the triangles surrounding a specific vertex. The SA is a trait with a high degree of heritability [33], and has been associated with cognitive abilities [34]. We determined cortical volume by multiplying the values of CT and SA. We averaged the values of these morphometric features (CT, SA and CV) for the parceled ROIs, as defined in the atlas provided by Desikan and colleagues (2006) and corrected for multiple comparison.

#### Network-based analysis of cortical and subcortical regions

We employed the processing pipeline introduced by Larivière et al. [16] to identify distinctive structural connectivity profiles exhibiting a significant correlation with morphometric abnormality maps. For this purpose, we used both CT and SA values to pinpoint regions in the cortex and subcortical structures. Regions were considered as epicenters due to their strong connections with regions showing high morphometric reductions and weaker connections with areas displaying lower levels of morphometric reductions.

The pipeline we used to identify these epicenters encompassed the following steps (detailed in https://enigma-toolbox.readthedocs.io): loading *z*-value maps for CT and SA, calculated as previously outlined; loading high-resolution structural (derived from diffusion-weighted tractography) connectivity matrix from a group of healthy adults in the Human Connectome Project, with parcellation based on Desikan et al. (2006); extracting seed-based structural connectivity matrices, utilizing each cortical and subcortical region as a seed.

#### Volumetric analysis of subcortical structures

We employed advanced computer-based modules for the subfield segmentations of subcortical structures. We adopted the method by Iglesias and colleagues [35] to provide an in-deep analysis of the hippocampal subfields. For the segmentation of amygdala nuclei, we adopted the method by Saygin et al. [36]. Such algorithms were optimized by these authors for subfield segmentations by adopting a computational atlas with a novel atlas-building algorithm based on Bayesian inference and working on T1-weighted images. To obtain more reliable segmentations, we included additional 3D T2-FLAIR MRIs in the processing pipeline. We finally inspected the subfield segmentations by visual analysis and manually corrected it where appropriate. For each subject, we calculated the volume of the segmented substructures of the hippocampus and amygdala.

#### Shape analysis of hippocampus and Cornu Ammonis

We used the segmentations obtained from the volumetric analysis to perform a shape analysis of the hippocampus and CA. We first considered the segmented masks and built the corresponding mesh of points using a MATLAB script (R2020b, MathWorks Inc., Natick, MA). We used the segmented volumes of the hippocampus and CA as inputs of SPHARM-MAT (https://www.nitrc.org/projects/spharm-mat), a tool based on a 3D Fourier surface representation method (SPHARM) that creates parametric surface models using the spherical harmonics [37]. The SPHARM processing pipeline included several steps. We pre-processed data to remove noises, therefore the 3D binary object presented a surface with a spherical topology. We repaired small vacancies and discontinuities between image voxels using the TopologyFix tool. We then adopted a parameterization algorithm to create a continuous and uniform mapping from the object surface to the surface of a unit sphere. We applied the CALD (control of area and length distortion) algorithm [38] to generate triangular meshes, consisting of vertices and faces. Then, with the SPHARM tool, we expanded the object surface into a complete set of spherical harmonic basis functions, which was a Fourier basis function defined on the sphere. By solving a linear system, we reconstructed the surface object using the Fourier coefficients up to a user-desired degree. Finally, with the SPHARM registration, we created a shape descriptor from a normalized set of SPHARM coefficients, which were comparable across objects for providing a group analysis. The registration used the first order ellipsoid for establishing surface correspondence and aligning objects. We applied surface-reconstruction steps for both the hippocampus and CA of both hemispheres.

To perform a comparison between patients and controls, we aligned models and carried a translation in the same center of reference using a high-resolution mesh (a six-level icosahendral mesh with 40962 vertices and 81920 faces). The same rotation matrix was used to register the CA in the same centroid with respect to the hippocampus and report the model in the center of the reference system.

### Statistical analysis

We carried out statistical analyses on volumetric features using FreeSurfer [28] and FSL [39] tools and MATLAB R2020b (Statistics and Machine Learning Toolbox Version 11.6, MathWorks Inc., Natick, MA).

#### Morphometric and volumetric analysis of cortical and subcortical regions

To evaluate the effect of the diagnostic group (*Group*) on morphometric measurements, we performed a group analysis between *PCDH19* patients and controls. To this purpose and mitigate confounding effects, we adopted a multivariate linear regression model using the center (*Center*) and age (*Age*) as covariates: ${Morph}={\beta }_{0}+{\beta }_{1}{Center}+{\beta }_{2}{Group}+{\beta }_{3}{Age}$. For volumetric measurements, we included in the model the estimated intra-cranial volume (*β*_4_*eICV*) as covariate.

#### Network-based analysis of cortical and subcortical regions

For each ROI, we evaluated the spatial correlation (*r*_*value*_) between the structural connectivity matrices and the *z*-value morphometry maps. We tested the statistical significance of each spatial correlation between seed-based cortico-cortical and subcortico-cortical connectivity and morphometric measurements using spin permutation tests (with 1,000 rotations and a significance threshold of *p*_value_ < 0.1).

#### Relationship between morphometric analysis e gene-expression patterns

For each ROI, we extracted the spatial PCDH19-expression levels (*ε*_value_) from the Allen Human Brain Atlas [18], a brain-wide gene expression atlas that includes measurement obtained through microarray technology from more than 20,000 genes, sampled across 3,702 spatially distinct tissue samples [40]. We calculated the spatial correlation (*r*_value_) between the PCDH19-expression levels (*ε*_value_) and morphometry features representing the structural SA and CT alterations (*β*_value_) and assessed significance testing by spin permutation tests (with 1,000 rotations and a significance threshold of *p*_spin_ < 0.1).

#### Relationship between morphometric and volumetric features and clinical findings

Maintaining the center and age as covariates, for each ROI, we estimated the effect of the morphometric and volumetric features on the clinical variables of patients using the following linear regression model: ${Clinics}={\beta }_{0}+{\beta }_{1}{Center}+{\beta }_{2}{Morph}+{\beta }_{3}{Age}$ (with *Clinics* as *Age at seizure onset*, *Cognitive level*, *Psychiatric disorder, Habitual seizures, number of treatments*).

#### Shape analysis of hippocampus and Cornu Ammonis

For the shape analysis, we assessed the difference between the mean shape of the two groups and calculated a *t*-test on the distribution of vertices of the volumetric meshes. We finally adopted a multivariate analysis of the covariance model (MANCOVA) to assess the covariance between the tridimensional meshes of the hippocampal shapes for each side.

## Results

### MRI visual analysis and clinical assessment

**Fig. 1:**
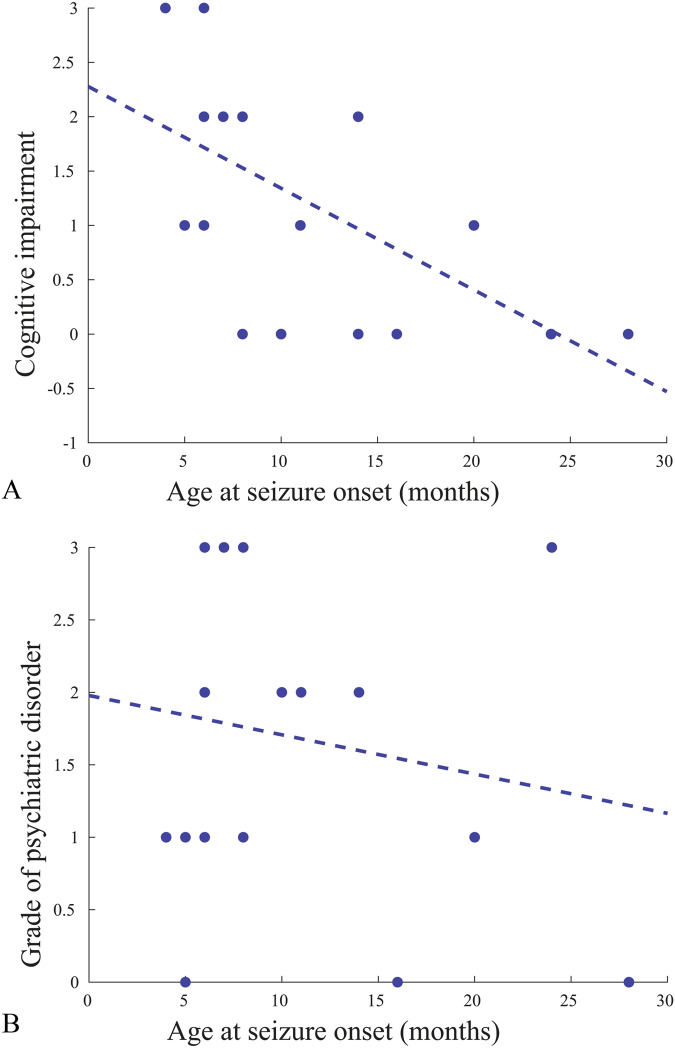
Correlation patterns in clinical variables. Correlation patterns between age at seizure onset and cognitive level (**A**) and severity of psychiatric disorder (**B**) in the cohort of patients with *PCDH19-*gene-related syndrome. Cognitive level was classified as follow: (0) “normal” or (1) “mild ID” or (2) “moderate ID” or (3) “severe ID”. Clinical values are summarized in Table 1.

After visual inspection of diagnostic MRI sequences (Supplementary Fig. 1 and Table 1), one patient (no. 16) was excluded from the volumetric analysis due to movement artifacts, and another patient (no. 10) was excluded from the hippocampal analysis as an incomplete inversion of the left hippocampus was observed on the MRI scan (Supplementary Fig. 2). We assessed associations between clinical variables (Fig. 1) and found that an earlier age at seizure onset was significantly correlated with cognitive impairment (*r*_value_ = -0.58, *p*_value_ = 0.008). The severity of psychiatric comorbidities did not show a significant correlation with age at seizure onset (*r*_value_ = -0.11, *p*_value_ = 0.640).

### Morphometric analysis of cortical regions

**Fig. 2:**
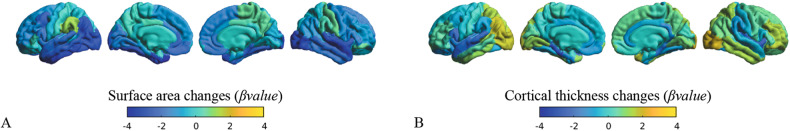
Morphometric analysis. Statistical ROI-based morphometric analysis of PCDH19-patients compared with matched controls. The results of the regression analysis on morphometric features, surface area (SA, **A**) and cortical thickness (CT, **B**), are represented by *β*_2_ maps (specified in Supplementary Table 1) superimposed on the *pial* surfaces of the left and right hemispheres. We observed statistically significant lower values of SA in left and right temporo-limbic cortices (blue ROIs, **A**), and normal SA in the surrounding regions (green ROIs, panel **A**). CT was significantly increased in right lateral occipital gyrus and parahippocampal regions bilaterally (yellow ROIs, **B**).

In morphometric analysis, as shown in Supplementary Table 1 and depicted in Fig. 2, we observed a significant reduction of SA in cortical areas of the limbic circuit in *PCDH19* patients compared to controls. ROIs with reduced SA were bilaterally the entorhinal (left/right *p*_value_ = 0.002/0.327), parahippocampal (left/right *p*_value_ = 0.230/0.016), fusiform (left/right *p*_value_ = 0.004/0.013), inferior temporal (left/right *p*_value_ = 0.002/0.062), superior temporal (left/right *p*_value_ = 0.029/0.003), rostral-anterior cingulate (left/right *p*_value_ = 0.018/0.069), medial orbito-frontal (left/right *p*_value_ = 0.034/0.055) gyri, temporal pole (left/right *p*_value_ = 0.001/0.004) and insula (left/right *p*_*value*_ = 0.067/0.006). The reduction of SA contributed to a proportional reduction of CV within the same regions. A reduced CT was noted only in the parahippocampal gyri (left/right *p*_value_ = 0.004/0.049).

### Network-based analysis of cortical and subcortical regions

**Fig. 3:**
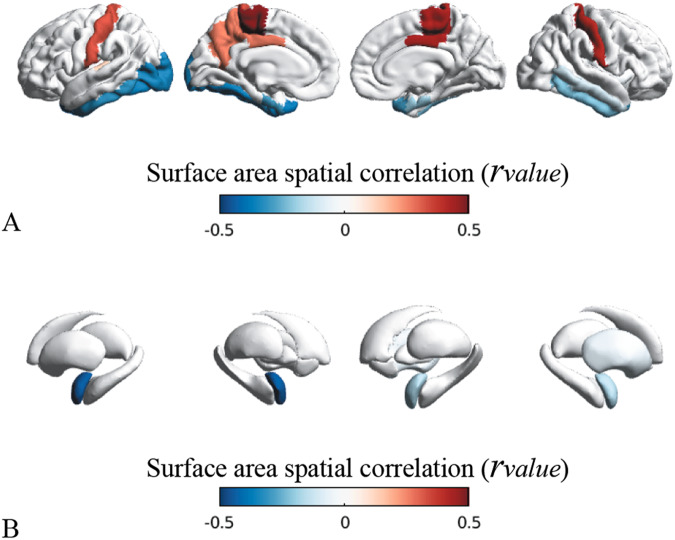
Network-based structural analysis of surface area (SA). Spatial correlations (*r*_value_, blue-red colormap) between SA patterns (*β*_2_) and seed-based cortico-cortical (**A**) and subcortico-cortical (**B**) connectivity profiles. In this example, we used as seed the left entorhinal gyrus. Correlation sco*r*es (*r*_value_) are detailed in Supplementary Tables 2 and 3. SA changes (*β*_2_) are indicated in Supplementary Table 1.

Based on the results of structural morphometric analysis, we conducted a network-based analysis of SA and observed a statistically significant structural correlation in the limbic regions (as depicted in Fig. 3). Regions where emerged a significant negative correlation (SA reduction, for details see Supplementary Tables 2 and 3) were the fusiform (left/right *p*_spin_ = 0.025/0.096), entorhinal (left/right *p*_spin_ = 0.073/0.034), parahippocampal (left/right *p*_spin_ = 0.230/0.016), inferior temporal (left/right *p*_spin_ = 0.014/0.036) gyri, temporal pole (left/right *p*_spin_ = 0.018/0.015), right insula (*p*_spin_ = 0.074) and amygdala (left/right *p*_spin_ = 0.003/0.033). We also noticed a positive correlation (SA increase) in paracentral (left/right *p*_spin_ = 0.004/0.006), postcentral (left/right *p*_spin_ = 0.030/0.009) and posterior cingulate (left/right *p*_*spin*_ = 0.089/0.013) gyri.

The network-based analysis of CT revealed no significant structural correlations (Supplementary Fig. 3 and Supplementary Tables 2 and 3).

### Relationship between structural morphometric analysis and gene-expression

**Fig. 4:**
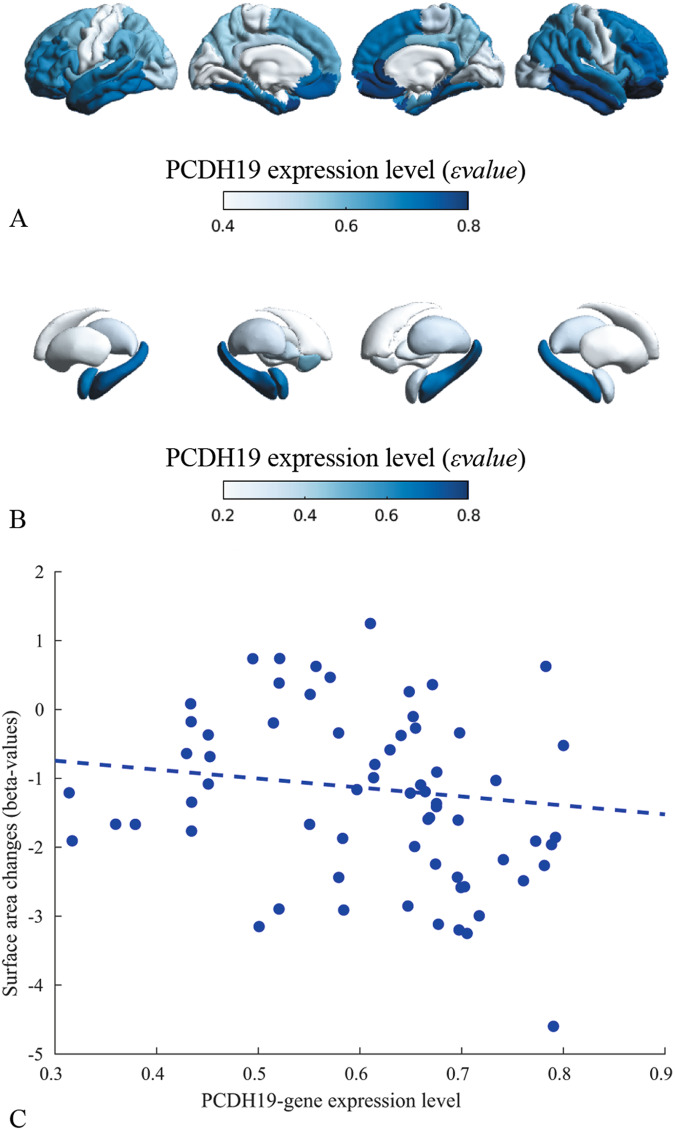
PCDH19-expression patterns and relationship with structural morphometric analysis. Spatial PCDH19 expression patterns (*ε*_*value*_) in cortical (**A**) and subcortical (**B**) regions of interest and (**C**) correlation between levels of PCDH19 expression and surface area changes (*β*_2_ detailed in Supplementary Table 1) observed in cortical and subcortical regions. The statistical correlation highlighted that regions with higher level of PCDH19 expression exhibited significantly higher reductions in SA (*r*_*value*_ = -0.262, *p*_*spin*_ = 0.092).

For each cortical and subcortical ROI, we extracted the spatial gene-expression coefficients (*ε*_*value*_) from the Allen Human Brain Atlas [18] (Fig. 4). *PCDH19* is significantly expressed in the cortical regions of the limbic circuit (Fig. 4A), in particular the fusiform (left/right *ε*_value_ = 0.717/0.701), entorhinal (left/right *ε*_value_ = 0.717/0.613), inferior temporal (left/right *ε*_value_ = 0.705/0.773), superior-temporal (left/right *ε*_value_ = 0.675/0.677), medial orbito-frontal (left/right *ε*_value_ = 0.741/0.788), and rostral-anterior cingulate (left/right *ε*_value_ = 0.696/0.792) gyri and temporal pole (left *ε*_value_ = 0.790). Among the limbic circuit, amygdala (left/right *ε*_value_ = 0.645/0.330) and hippocampus (left/right *ε*_value_ = 0.689/0.664) are the subcortical regions with the highest PCDH19 expression (Fig. 4B). We also noticed that cortical regions with positive correlations in network-based analysis (SA increase) had lower PCDH19 expression levels, i.e. paracentral (left/right *ε*_value_ = 0.434/0.494), postcentral (left/right *ε*_value_ = 0.433/0.521) and posterior cingulate (left/right *ε*_value_ = 0.520/0.570) gyri.

We correlated morphometric alterations in the cortical and subcortical regions with the levels of PCDH19 expression (Fig. 4C) and observed that regions with highest PCDH19 levels had a statistically significant reduction of SA (*r*_value_ = -0.262, *p*_spin_ = 0.092). Subcortical regions exhibited no volumetric reductions, hence no correlations between volume reduction and expression level. We observed no statistically significant correlations between gene-expression levels and CT alterations (*r*_value_ = -0.127, *p*_spin_ = 0.185, Supplementary Fig. 4).

### Relationship between morphometric analysis and severity of psychiatric comorbidities, habitual seizures and treatments

**Fig. 5:**
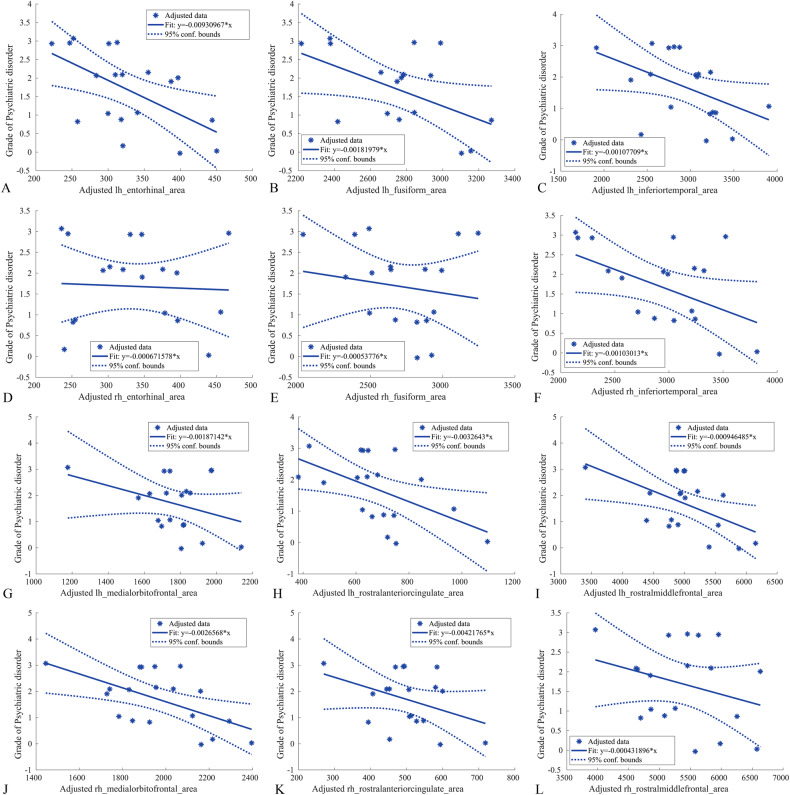
Grade of psychiatric comorbidities and relationship with structural morphometric analysis. Significant regression patterns between the severity of psychiatric comorbidities and values of surface area (SA) of a representative subset of the cortical regions (left and right entorhinal, **A** and **D**; left and right fusiform, **B** and **E**; left and right inferiortemporal, **C** and **F**; left and right medialorbitofrontal, **G** and **J**; left and right rostralanteriorcingulate, **H** and **K**; left and right rostralmiddlefrontal, **I** and **L**, as reported in Supplementary Table 4) in *PCDH19*-mutated patients. The values, indicated by asterisks, are adjusted according to the linear regression model applied in the statistical analysis. The continuous line represents the fitting line, the dotted line indicated the 95% confidence bounds. We observed a negative trend in the fitting lines, that show a reduced SA in patients with more severe psychiatric disorder. Legend: lh, left hemisphere; rh, right hemisphere.

The effect of morphometric variables on psychiatric comorbidities (summarized in Table 1) was significant in several areas of the limbic cortex (Supplementary Table 4). Patients with more severe psychiatric symptoms exhibited lower values of SA in the left entorhinal (*β*_*2*_ = -0.0093, *p*_value_ = 0.013), fusiform (*β*_*2*_ = -0.0018, *p*_value_ = 0.046), inferior temporal (*β*_*2*_ = -0.0011, *p*_value_ = 0.013), rostral anterior cingulate (*β*_*2*_ = -0.0033, *p*_value_ = 0.025), rostral middle frontal (*β*_*2*_ = -0.0009, *p*_value_ = 0.022) and right medial orbito-frontal (*β*_*2*_ = -0.0027, *p*_value_ = 0.013) gyri (see Fig. 5). The effect on cortical thickness of habitual seizures was significant in two regions (Supplementary Table 5), the left caudal anterior (*β*_*2*_ = 1.1745, *p*_value_ = 0.008) and posterior (*β*_*2*_ = 1.5761, *p*_value_ = 0.008) cingulate. The pharmacological load (number of treatments, Supplementary Table 6) was significant for the cortical thickness of the entorhinal regions (left/right *β*_*2*_ = 1.4803/1.8152, *p*_value_ = 0.014/0.010).

### Analysis of subfields in subcortical structures

The group analysis of subcortical structures revealed volumetric reductions involving the hippocampal subfields bilaterally in the *PCDH19* group compared to controls (Supplementary Table 7 and Supplementary Fig. 5). We observed a statistically significant volumetric reduction in the right hippocampal tail (*p*_value_ = 0.048), hippocampal fissure (left/right *p*_value_ = 0.003/0.014), right body of molecular layer (*p*_value_ = 0.016), right GC-ML-DG region (*p*_value_ = 0.007), head of the left CA3 region (*p*_value_ = 0.002), body of the right CA3 (*p*_value_ = 0.004), and of the right CA4 (*p*_value_ = 0.011). The volume of other segments of CA were also reduced, although not reaching statistically significant values (body of CA1 left/right *p*_value_ = 0.103/0.089; right CA3: *p*_value_ = 0.084; body of the left CA3: *p*_value_ = 0.078; head of the CA4: left/right *p*_value_ = 0.115/0.119; body of the left CA4: *p*_value_ = 0.083). We found that the volume of the body of the right hippocampus was significantly reduced (*p*_value_ = 0.026), while the whole hippocampus was also reduced, but not significantly (left/right *p*_value_ = 0.330/0.146), as we had previously measured [6].

In the left amygdala subunits (Supplementary Table 8), we found not significantly reduced values in central (*p*_value_ = 0.100), medial (*p*_value_ = 0.100) and cortical (*p*_value_ = 0.082) nuclei, in patients with respect to controls.

### Relationship between volume of subfields in subcortical structures and clinical findings

When we correlated the volumetric values with clinical variables (summarized in Table 1), we observed that patients with more severe psychiatric comorbidities exhibited lower volumetric values (Supplementary Table 9 and Supplementary Fig. 6), in particular in the heads of right CA3 (*β*_*2*_ = -0.0278, *p*_value_ = 0.049) and left CA4 (*β*_*2*_ = -0.0302, *p*_value_ = 0.038) and body of the right hippocampus (*β*_*2*_ = -0.0045, *p*_value_ = 0.048). We observed no correlation between hippocampal volumetric assessments and age at seizure onset or cognitive level.

### Shape analysis of hippocampus and Cornu Ammonis

We evaluated the mean difference between the shape of groups of *PCDH19* patients and controls (Supplementary Fig. 7) by a *t*-test with a corresponding statistical significance (*p*_*value*_, Supplementary Fig. 8) and observed that the shape of the hippocampus was moderately different in several regions (Supplementary Fig. 8B and C). The main regions of significance in our comparative analyses were primarily located in the CA (Supplementary Fig. 9), which exhibited abnormal shapes in *PCDH19* patients with respect to controls.

## Discussion

In this cohort of patients with *PCDH19* mutations we observed a significant correlation between age at seizure onset and ID, consistently with suggestions that a causal relationship exists in these genetic disorder between early onset seizures and adverse cognitive outcomes (Kolc et al. [3]).

In the morphometric analysis of the cortex, we observed *PCDH19* patients to exhibit bilateral SA reductions in the cortical regions forming the limbic circuit, i.e. fusiform, entorhinal, parahippocampal, inferior and superior temporal, medial orbito-frontal and rostral-anterior cingulate gyri, temporal pole and insula. In our network-based analysis of SA the amygdala emerged as a structural epicenter in correlation with the abnormal patterns in cortical limbic regions. Morphometric and network-based analysis strengthen our previous surface-based findings [6], revealing clusters of reduced gyrification index in the limbic regions. Cortical gyrification represents the culmination of complex growth mechanisms influenced by genetic, molecular factors, and mechanical forces, initiating during fetal development and persisting throughout life [41, 42]. Cortical SA is proportional to the gyrification index [43], since the process of gyrification aids in augmenting the SA within finite spatial confines, and potentially enhances connectivity by reducing inter-regional distances [44]. SA is a highly heritable trait, yet little is known about genetic influences on regional cortical differentiation in humans [45, 46]. From the third trimester of pregnancy to the second year of age, the cortex expands dynamically in a regionally heterogeneous fashion [47]. SA is the result of such mechanisms of expansion [48]. We observed that reduced SA values significantly correlated with post-natal PCDH19-expression levels as reported in the atlas of the human brain transcriptome [40]. More precise correlations would be possible also considering prenatal PCDH19 expression levels, but this information is not fully available in public databases. We interrogated the Mouse Genome Informatics (MGI) database (https://www.informatics.jax.org/) and extracted PCDH19 expression levels (Supplementary Table 10), from embryonic day 0 (E0) to birth (postnatal day 0, P0). In mouse embryo, PCDH19 is expressed since early brain development (E8.5) and its expression has been detected at different stages in three regions participating to the limbic circuit, cerebral cortex (from E11.5 to E15.5), hippocampus (from E14.5 to E15.5), and thalamus (E18.5). In the mouse, E8.5 falls in the neurulation stage, the time windows from E11.5 to E15.5 and E18.5 are included in neurogenesis, neuronal proliferation and migration, and dendritic development and synaptogenesis [49]. Thus, developmental brain anomalies observed in patients carrying *PCDH19* mutations might occur both during prenatal stages and postnatal maturation processes.

We quantitatively examined the volume and shape of the hippocampus, exploring all its components, and found patients to exhibit highly significant reductions in the volumes and shape differences in several subunits, particularly the CA. Studies exploring quantitative subfield alterations in TLE reported an overall effect of temporal lobe seizures manifesting as bilateral medial hippocampal atrophy, and a more selective effect of hippocampal seizures, leading to disease-proportional CA1 atrophy [50, 51]. The hippocampal formation acts as an epicenter of more intense structural changes even in epilepsy syndromes in which no clear indication of seizure genesis in the hippocampus exists, such as *SCN1A*-related Dravet syndrome [52] and idiopathic generalized epilepsy syndromes [20, 53]. In a previous study of *PCDH19*-related encephalopathy, we demonstrated altered gyrification in the parahippocampal and entorhinal regions, and diffusion abnormalities in the underlying white matter [6], but no alterations emerged in the hippocampus when it was examined as a whole. The novel findings we present in this study demonstrate that specific hippocampal subfields show significantly reduced volumes to an extent that did not emerge when analyzing the whole hippocampus.

We suppose that structural alterations in the hippocampus, amygdala and connected cortical regions of the limbic circuit might be the expression of specific *PCDH19* defects underlying irregular growth dynamics. In-vitro studies and experimental animal models corroborate this hypothesis, since *PCDH19* expression patterns play a key role in the formation of limbic regions [21, 54], more specifically the CA [22]. The PCDH19 protein is also abundant in the mossy fiber pathway [55], where its expression levels increase postnatally during synapse development [56]. The mossy fiber pathway terminates in synapses formed by dentate granule cells onto CA3 pyramidal neurons. *Pcdh19* mRNA is expressed by both dentate granule cells and CA3 pyramidal neurons, indicating that *PCDH19* regulates the development, stabilization, maintenance and function of both the pre- and post-synaptic neurons of mossy fiber synapses [13]. *PCDH19* mutations might also impair columnar organization [57] and lead to deviations in developmental trajectories at a network level [58]. Animal models have highlighted how *PCDH19* intervenes in regulating the formation and maintenance of brain networks [13], and how mosaic mutations disrupt local cortical networks [14]. Mouse models in which PCDH19 is expressed in mosaic fashion exhibit structural and functional synaptic abnormalities in the hippocampus, with impaired neuronal network activity and connectivity [15].

The limbic cortex and amygdala emerged as structural epicenters of SA alterations, i.e. regions strongly inter-connected with regions in which reduced SA emerged, and weakly inter-connected with regions with normal SA. Structural abnormalities within the limbic network may correspond to specific clinical manifestations in individuals with *PCDH19* encephalopathy as suggested by the frequently observed pattern of focal seizures with affective symptoms [1], also confirmed in most patients within the current cohort. We also evaluated the effect on morphometry of pharmacological load and habitual seizures and found no significant effect at either the SA or cortical thickness globally, while the left caudal cingulate and entorhinal cortex revealed a weak correlation with active epilepsy.

*PCDH19-*related epilepsies are among the eight most common single-gene epilepsies, with an incidence of 1 per 20,600 live-born females, and a prevalence of 4.85/100,000 (95% CI 1.97–9.15) [59]. Conditions resulting from single-gene mutations provide an adequate paradigm for correlating specific imaging changes with behavioral and psychiatric phenotypes. Our observations indicate that patients exhibiting more severe psychiatric manifestations had more prominent pattern of anatomic aberration, notably involving the entorhinal and fusiform cortex, hippocampal body and CA3. Such findings align with our previous study demonstrating that in PCDH19 encephalopathy, more significant gyrification and diffusion abnormalities in the mesio-temporal regions occur in individuals with ASD [6]. A correlation between atypical mesio-temporal anatomy and autism has been demonstrated using MRI in various clinical contexts [7–10]. Post-mortem brain studies in individuals with autism have revealed abnormalities in the hippocampus, amygdala and connected limbic structures [60, 61]. Altered amygdala development has been demonstrated in children with autism [62, 63], but little is known about the simultaneous development of the brain regions directly connected with the amygdala [64]. Autism has been associated with genes related to the regulation of neuronal proliferation [65] and transcriptionally downregulated genes implicated in autism have been robustly associated with global changes in cortical thickness variability in children with autism [66]. Such findings corroborate our observations that the neurodevelopmental trajectory of brain maturation may be atypical in ASD [67], and that structural brain imaging offers clues about the effect of the genetic etiology of neuropsychiatric disorders [68].

Advanced neuroimaging holds great promise as a tool for categorizing patients with different neuropsychiatric manifestations [69] and may elucidate genotype-phenotype correlations at the patient level. Recent advances in machine learning, combined with quantitative neuroimaging methods, have demonstrated their remarkable capability to discern disparities in local morphological features across various brain subregions and elucidated variations between patients groups, a crucial step toward establishing a reliable psychiatric nosology, understanding disease progression and predicting individual disease trajectories [70]. MRI-based quantitative methods are a potential tool for early characterization and quantification of anatomical changes to predict the individual-level progression of clinical variables, such as the likelihood of experiencing cognitive disability and psychiatric comorbidities.

Although our methodological approaches have detected anatomic differences between patients with *PCDH19* encephalopathy and a control population, several limitations must be acknowledged. We only included 20 patients, with a wide age range. However, *PCDH19* encephalopathy remains rare, has its onset in the pediatric age and is associated with impaired cognitive function, which may pose challenges in systematically assessing psychiatric features. Since we analyzed images acquired from two different scanners, we adopted a multivariate statistical model to mitigate biases among centers. Furthermore, while analyzing the structural characteristics of hippocampal subfields presents challenges, the tools we applied have undergone extensive validation and have been adopted in numerous previous investigations [71]. Effects of genetic variant types (missense vs other) on morphometric features, expression patterns and clinical manifestations can be studied adopting suitable statistical methods for exploring data from larger cohorts [3].

### Supplementary information

Supplementary material

## Data Availability

Data are available from corresponding author upon reasonable request.

## References

[1] Marini C, Darra F, Specchio N, Mei D, Terracciano A, Parmeggiani L (2012). Focal seizures with affective symptoms are a major feature of PCDH19 gene-related epilepsy. Epilepsia.

[2] Rodriguez-Cruces R, Royer J, Larivière S, Bassett DS, Caciagli L, Bernhardt BC. Multimodal connectome biomarkers of cognitive and affective dysfunction in the common epilepsies. Netw Neurosci. 2022;6:320-38. 10.1162/netn_a_00237 PMC9208009 35733426

[3] Kolc KL, Sadleir LG, Scheffer IE, Ivancevic A, Roberts R, Pham DH (2019). A systematic review and meta-analysis of 271 PCDH19-variant individuals identifies psychiatric comorbidities, and association of seizure onset and disease severity. Mol Psychiatry.

[4] Trivisano M, Pietrafusa N, Terracciano A, Marini C, Mei D, Darra F (2018). Defining the electroclinical phenotype and outcome of PCDH19-related epilepsy: A multicenter study. Epilepsia.

[5] Kurian M, Korff CM, Ranza E, Bernasconi A, Lübbig A, Nangia S (2018). Focal cortical malformations in children with early infantile epilepsy and PCDH19 mutations: case report. Dev Med Child Neurol.

[6] Lenge M, Marini C, Canale E, Napolitano A, de Masi S, Trivisano M (2020). Quantitative MRI-based analysis identifies developmental limbic abnormalities in PCDH19 encephalopathy. Cereb Cortex.

[7] Travers BG, Adluru N, Ennis C, Tromp DPM, Destiche D, Doran S (2012). Diffusion Tensor Imaging in Autism Spectrum Disorder: A Review. Autism Res.

[8] Wallace GL, Robustelli B, Dankner N, Kenworthy L, Giedd JN, Martin A (2013). Increased gyrification, but comparable surface area in adolescents with autism spectrum disorders. Brain.

[9] Ecker C, Andrews D, Dell’Acqua F, Daly E, Murphy C, Catani M (2016). Relationship between cortical gyrification, white matter connectivity, and autism spectrum disorder. Cereb Cortex.

[10] Duret P, Samson F, Pinsard B, Barbeau EB, Boré A, Soulières I (2018). Gyrification changes are related to cognitive strengths in autism. NeuroImage Clin.

[11] Hazlett HC, Gu H, Munsell BC, Kim SH, Styner M, Wolff JJ (2017). Early brain development in infants at high risk for autism spectrum disorder. Nature.

[12] Gerosa L, Francolini M, Bassani S, Passafaro M (2019). The Role of Protocadherin 19 (PCDH19) in Neurodevelopment and in the Pathophysiology of Early Infantile Epileptic Encephalopathy-9 (EIEE9). Dev Neurobiol.

[13] Hoshina N, Johnson-Venkatesh EM, Hoshina M, Umemori H Female-specific synaptic dysfunction and cognitive impairment in a mouse model of PCDH19 disorder. Science (80-) 2021;372. 10.1126/science.aaz3893. 10.1126/science.aaz3893 PMC9873198 33859005

[14] Lamers D, Landi S, Mezzena R, Baroncelli L, Pillai V, Cruciani F (2022). Perturbation of Cortical Excitability in a Conditional Model of PCDH19 Disorder. Cells.

[15] Giansante G, Mazzoleni S, Zippo AG, Ponzoni L, Ghilardi A, Maiellano G et al. Neuronal network activity and connectivity are impaired in a conditional knockout mouse model with PCDH19 mosaic expression. Mol Psychiatry 2023. 10.1038/s41380-023-02022-1. 10.1038/s41380-023-02022-1 PMC11371655 36997609

[16] Larivière S, Rodríguez-Cruces R, Royer J, Caligiuri ME, Gambardella A, Concha L (2020). Network-based atrophy modeling in the common epilepsies: A worldwide ENIGMA study. Sci Adv.

[17] Larivière S, Royer J, Rodríguez-Cruces R, Paquola C, Caligiuri ME, Gambardella A (2022). Structural network alterations in focal and generalized epilepsy assessed in a worldwide ENIGMA study follow axes of epilepsy risk gene expression. Nat Commun.

[18] Arnatkevic̆iūtė A, Fulcher BD, Fornito A (2019). A practical guide to linking brain-wide gene expression and neuroimaging data. Neuroimage.

[19] Nees F, Pohlack ST (2014). Functional MRI studies of the hippocampus. Hippocampus Clin Neurosci.

[20] Whelan CD, Altmann A, Botía JA, Jahanshad N, Hibar DP, Absil J (2018). Structural brain abnormalities in the common epilepsies assessed in a worldwide ENIGMA study. Brain.

[21] Kim SY, Mo JW, Han S, Choi SY, Han SB, Moon BH (2010). The expression of non-clustered protocadherins in adult rat hippocampal formation and the connecting brain regions. Neuroscience.

[22] Kim SY, Chung HS, Sun W, Kim H (2007). Spatiotemporal expression pattern of non-clustered protocadherin family members in the developing rat brain. Neuroscience.

[23] Specchio N, Marini C, Terracciano A, Mei D, Trivisano M, Sicca F (2011). Spectrum of phenotypes in female patients with epilepsy due to protocadherin 19 mutations. Epilepsia.

[24] Cappelletti S, Specchio N, Moavero R, Terracciano A, Trivisano M, Pontrelli G (2015). Cognitive development in females with PCDH19 gene-related epilepsy. Epilepsy Behav.

[25] Harris PA, Taylor R, Thielke R, Payne J, Gonzalez N, Conde JG (2009). Research electronic data capture (REDCap)-A metadata-driven methodology and workflow process for providing translational research informatics support. J Biomed Inf.

[26] American Psychiatric Association. Diagnostic and Statistical Manual of Mental Disorders. American Psychiatric Association, 2013 10.1176/appi.books.9780890425596.

[27] Fischl B, Salat DH, Busa E, Albert M, Dieterich M, Haselgrove C (2002). Whole brain segmentation: Automated labeling of neuroanatomical structures in the human brain. Neuron.

[28] Fischl B (2012). FreeSurfer. Neuroimage.

[29] Fischl B, van der Kouwe A, Destrieux C, Halgren E, Ségonne F, Salat DH (2004). Automatically Parcellating the Human Cerebral Cortex. Cereb Cortex.

[30] Fischl B, Dale AM (2000). Measuring the thickness of the human cerebral cortex from magnetic resonance images. Proc Natl Acad Sci USA.

[31] Rakic P (1988). Specification of cerebral cortical areas. Science (80-).

[32] Huttenlocher PR (1990). Morphometric study of human cerebral cortex development. Neuropsychologia.

[33] Panizzon MS, Fennema-Notestine C, Eyler LT, Jernigan TL, Prom-Wormley E, Neale M (2009). Distinct Genetic Influences on Cortical Surface Area and Cortical Thickness. Cereb Cortex.

[34] Vuoksimaa E, Panizzon MS, Chen C-H, Fiecas M, Eyler LT, Fennema-Notestine C (2015). The Genetic Association Between Neocortical Volume and General Cognitive Ability Is Driven by Global Surface Area Rather Than Thickness. Cereb Cortex.

[35] Iglesias JE, Augustinack JC, Nguyen K, Player CM, Player A, Wright M (2015). A computational atlas of the hippocampal formation using ex vivo, ultra-high resolution MRI: Application to adaptive segmentation of in vivo MRI. Neuroimage.

[36] Saygin ZM, Kliemann D, Iglesias JE, van der Kouwe AJW, Boyd E, Reuter M (2017). High-resolution magnetic resonance imaging reveals nuclei of the human amygdala: manual segmentation to automatic atlas. Neuroimage.

[37] Brechbühler C, Gerig G, Kübler O (1995). Parametrization of closed surfaces for 3-D shape description. Comp Vis Graph Image Process.

[38] Shen L, Makedon F (2006). Spherical mapping for processing of 3D closed surfaces. Image Vis Comput.

[39] Jenkinson M, Beckmann CF, Behrens TEJJ, Woolrich MW, Smith SM (2012). Fsl. Neuroimage.

[40] Hawrylycz MJ, Lein ES, Guillozet-Bongaarts AL, Shen EH, Ng L, Miller JA (2012). An anatomically comprehensive atlas of the adult human brain transcriptome. Nature.

[41] Llinares-Benadero C, Borrell V (2019). Deconstructing cortical folding: genetic, cellular and mechanical determinants. Nat Rev Neurosci.

[42] Hogstrom LJ, Westlye LT, Walhovd KB, Fjell AM (2013). The structure of the cerebral cortex across adult life: Age-related patterns of surface area, thickness, and gyrification. Cereb Cortex.

[43] Schaer M, Cuadra MBB, Tamarit L, Lazeyras F, Eliez S, Thiran J (2008). A Surface-Based Approach to Quantify Local Cortical Gyrification. IEEE Trans Med Imaging.

[44] Docherty AR, Hagler DJ, Panizzon MS, Neale MC, Eyler LT, Fennema-Notestine C (2015). Does degree of gyrification underlie the phenotypic and genetic associations between cortical surface area and cognitive ability?. Neuroimage.

[45] Chen CH, Gutierrez ED, Thompson W, Panizzon MS, Jernigan TL, Eyler LT (2012). Hierarchical genetic organization of human cortical surface area. Science (80-).

[46] van der Meer D, Kaufmann T (2022). Mapping the genetic architecture of cortical morphology through neuroimaging: progress and perspectives. Transl Psychiatry.

[47] Huang Y, Wu Z, Wang F, Hu D, Li T, Guo L (2022). Mapping developmental regionalization and patterns of cortical surface area from 29 post-menstrual weeks to 2 years of age. Proc Natl Acad Sci.

[48] Lyall AE, Shi F, Geng X, Woolson S, Li G, Wang L (2015). Dynamic Development of Regional Cortical Thickness and Surface Area in Early Childhood. Cereb Cortex.

[49] Chini M, Hanganu-Opatz IL (2021). Prefrontal Cortex Development in Health and Disease: Lessons from Rodents and Humans. Trends Neurosci.

[50] Maccotta L, Moseley ED, Benzinger TL, Hogan RE (2015). Beyond the CA1 subfield: Local hippocampal shape changes in MRI-negative temporal lobe epilepsy. Epilepsia.

[51] Hogan RE, Moseley ED, Maccotta L (2015). Hippocampal Surface Deformation Accuracy in T1-Weighted Volumetric MRI Sequences in Subjects with Epilepsy. J Neuroimaging.

[52] Lenge M, Balestrini S, Mei D, Macconi L, Caligiuri ME, Cuccarini V (2023). Morphometry and network-based atrophy patterns in SCN1A-related Dravet syndrome. Cereb Cortex.

[53] Bouyeure A, Patil S, Mauconduit F, Poiret C, Isai D, Noulhiane M (2021). Hippocampal subfield volumes and memory discrimination in the developing brain. Hippocampus.

[54] Hirano S, Yan Q, Suzuki ST (1999). Expression of a novel protocadherin, OL-protocadherin, in a subset of functional systems of the developing mouse brain. J Neurosci.

[55] Schaarschuch A, Hertel N (2018). Expression profile of N-cadherin and protocadherin-19 in postnatal mouse limbic structures. J Comp Neurol.

[56] Terauchi A, Johnson-Venkatesh EM, Toth AB, Javed D, Sutton MA, Umemori H (2010). Distinct FGFs promote differentiation of excitatory and inhibitory synapses. Nature.

[57] Cooper SR, Emond MR, Duy PQ, Liebau BG, Wolman MA, Jontes JD (2015). Protocadherins control the modular assembly of neuronal columns in the zebrafish optic tectum. J Cell Biol.

[58] Light SEW, Jontes JD (2019). Multiplane calcium imaging reveals disrupted development of network topology in zebrafish pcdh19 mutants. eNeuro.

[59] Symonds JD, Zuberi SM, Stewart K, Mclellan A, O’regan M, Macleod S (2019). Molecular epidemiology of monogenic epilepsies answers key clinical questions. Brain.

[60] Ben Shalom D (2003). Memory in autism: Review and synthesis. Cortex.

[61] Bauman ML, Kemper TL (2005). Neuroanatomic observations of the brain in autism: A review and future directions. Int J Dev Neurosci.

[62] Amaral DG, Nordahl CW (2022). Amygdala Involvement in Autism: Early Postnatal Changes, But What Are the Behavioral Consequences?. Am J Psychiatry.

[63] Shen MD, Swanson MR, Wolff JJ, Elison JT, Girault JB, Kim SH (2022). Subcortical Brain Development in Autism and Fragile X Syndrome: Evidence for Dynamic, Age- and Disorder-Specific Trajectories in Infancy. Am J Psychiatry.

[64] Lee JK, Andrews DS, Ozturk A, Solomon M, Rogers S, Amaral DG (2022). Altered Development of Amygdala-Connected Brain Regions in Males and Females with Autism. J Neurosci.

[65] Stein JL, de la Torre-Ubieta L, Tian Y, Parikshak NN, Hernández IA, Marchetto MC (2014). A quantitative framework to evaluate modeling of cortical development by neural stem cells. Neuron.

[66] Romero-Garcia R, Warrier V, Bullmore ET, Baron-Cohen S, Bethlehem RAI (2019). Synaptic and transcriptionally downregulated genes are associated with cortical thickness differences in autism. Mol Psychiatry.

[67] Ecker C, Bookheimer SY, Murphy DGM (2015). Neuroimaging in autism spectrum disorder: brain structure and function across the lifespan. Lancet Neurol.

[68] Radonjić NV, Hess JL, Rovira P, Andreassen O, Buitelaar JK, Ching CRK (2021). Structural brain imaging studies offer clues about the effects of the shared genetic etiology among neuropsychiatric disorders. Mol Psychiatry.

[69] Bahathiq RA, Banjar H, Bamaga AK, Jarraya SK Machine learning for autism spectrum disorder diagnosis using structural magnetic resonance imaging: Promising but challenging. Front Neuroinform 2022;16. 10.3389/fninf.2022.949926. 10.3389/fninf.2022.949926 PMC9554556 36246393

[70] Yassin W, Nakatani H, Zhu Y, Kojima M, Owada K, Kuwabara H et al. Machine-learning classification using neuroimaging data in schizophrenia, autism, ultra-high risk and first-episode psychosis. Transl Psychiatry 2020;10. 10.1038/s41398-020-00965-5. 10.1038/s41398-020-00965-5 PMC7429957 32801298

[71] Sämann PG, Iglesias JE, Gutman B, Grotegerd D, Leenings R, Flint C et al. FreeSurfer-based segmentation of hippocampal subfields: A review of methods and applications, with a novel quality control procedure for ENIGMA studies and other collaborative efforts. Hum. Brain Mapp. 2020. 10.1002/hbm.25326. 10.1002/hbm.25326 PMC8805696 33368865

