# Supplementary material for: Morphometric network-based abnormalities correlate with psychiatric comorbidities and gene expression in PCDH19-related developmental and epileptic encephalopathy
Source: Transl Psychiatry. 2024 Jan 18;14:35. doi: 10.1038/s41398-024-02753-x (PMC10796344; doi:10.1038/s41398-024-02753-x)
Supplement: Supplementary file 1 — Supplementary material [file 41398_2024_2753_MOESM1_ESM.docx]

**Supplementary Information**

**Morphometric network-based abnormalities correlate with psychiatric comorbidities and gene expression in *PCDH19-*related developmental and epileptic encephalopathy**

Matteo Lenge^1, *^ PhD, Simona Balestrini^1, *^ PhD, Antonio Napolitano^2, *^ PhD, Davide Mei^1^ MSc, Valerio Conti^1^ PhD, Giulia Baldassarri^2^ MSc, Marina Trivisano^3^ PhD, Simona Pellacani^1^ MD, Letizia Macconi^4^ MD, Daniela Longo^5^ MD, Maria Camilla Rossi Espagnet^5^ PhD, Simona Cappelletti^3^ PsyD, PCDH19 Clinical Study Group^§^, Ludovico D’Incerti^4^ MD, Carmen Barba^1^ PhD, Nicola Specchio^3^ PhD, and Renzo Guerrini^1^ MD, FRCP, FAES

^1^ Child Neurology Unit and Laboratories, Neuroscience Department, Meyer Children’s Hospital IRCCS, 50139 Florence, Italy

^2^ Medical Physics Department, Bambino Gesù Children's Hospital, IRCCS, 00100 Rome, Italy

^3^ Neurology, Epilepsy and Movement Disorders, Bambino Gesù Children’s Hospital, IRCCS, Full Member of European Reference Network EpiCARE, 00165 Rome, Italy

^4^ Pediatric Radiology Unit, Meyer Children’s Hospital IRCCS, 50139 Florence, Italy

^5^ Functional and Interventional Neuroimaging Unit, Bambino Gesù Children’s Hospital, IRCCS, 00165 Rome, Italy

^§^ See Appendix for PCDH19 Clinical Study Group collaborators.

^*^ These authors contributed equally to the work.

Correspondence to:

Prof. Renzo Guerrini MD, FRCP, FAES

Neuroscience Department, Children’s Hospital A. Meyer IRCCS - University of Florence, Viale Pieraccini 24, 50139 Firenze, Italy. Tel: +390555662573; Fax: +390555662329; E-mail: [renzo.guerrini@meyer.it](mailto:renzo.guerrini@meyer.it)

| **Supplementary Table 1. Morphometric region-of-interest-based analysis** | | | | | | | | | | |
| --- | --- | --- | --- | --- | --- | --- | --- | --- | --- | --- |
|  |  |  | Morphometric features | | | | | | | |
|  |  |  | Surface  Area [mm^2^] | |  | Cortical  Thickness [mm] | |  | Cortical  Volume [mm^3^] | |
| **Cortical region** | **Side** |  | $\beta_{2}$ | ***p*_value_** |  | $\beta_{2}$ | ***p*_value_** |  | $\beta_{2}$ | ***p*_value_** |
| Bankssts | L |  | -0.798 | 0.428 |  | 1.213 | 0.231 |  | -0.270 | 0.788 |
|  | R |  | -1.096 | 0.278 |  | 0.017 | 0.987 |  | -0.997 | 0.324 |
| Caudal anterior cingulate | L |  | -1.667 | 0.101 |  | -0.452 | 0.653 |  | -1.703 | 0.095 |
|  | R |  | -0.909 | 0.368 |  | -1.023 | 0.311 |  | -1.797 | 0.078 |
| Caudal middle frontal | L |  | **-2.438** | **0.018** |  | -0.756 | 0.453 |  | **-2.441** | **0.018** |
|  | R |  | -1.365 | 0.178 |  | 0.638 | 0.526 |  | -1.257 | 0.214 |
| Cuneus | L |  | -1.764 | 0.084 |  | 0.580 | 0.564 |  | -1.312 | 0.195 |
|  | R |  | -0.684 | 0.497 |  | 0.191 | 0.849 |  | -0.811 | 0.421 |
| Entorhinal | L |  | **-3.200** | **0.002** |  | -1.480 | 0.145 |  | **-3.451** | **0.001** |
|  | R |  | -0.990 | 0.327 |  | -0.334 | 0.740 |  | -0.833 | 0.408 |
| Fusiform | L |  | **-2.994** | **0.004** |  | -0.799 | 0.428 |  | **-3.141** | **0.003** |
|  | R |  | **-2.573** | **0.013** |  | -1.042 | 0.302 |  | **-2.826** | **0.007** |
| Inferior parietal | L |  | **-2.911** | **0.005** |  | 1.795 | 0.078 |  | -1.611 | 0.113 |
|  | R |  | -1.193 | 0.238 |  | 0.785 | 0.436 |  | -0.882 | 0.382 |
| Inferior temporal | L |  | **-3.250** | **0.002** |  | 1.182 | 0.243 |  | **-2.367** | **0.022** |
|  | R |  | -1.911 | 0.062 |  | 1.317 | 0.194 |  | -0.863 | 0.392 |
| Isthmus cingulate | L |  | -0.196 | 0.846 |  | -0.341 | 0.735 |  | -0.853 | 0.398 |
|  | R |  | -0.103 | 0.918 |  | -1.491 | 0.142 |  | -1.516 | 0.136 |
| Lateral occipital | L |  | **-2.896** | **0.006** |  | 1.719 | 0.092 |  | -1.781 | 0.081 |
|  | R |  | **-3.151** | **0.003** |  | **2.040** | **0.046** |  | -1.883 | 0.065 |
| Lateral orbito-frontal | L |  | -0.339 | 0.736 |  | -1.392 | 0.170 |  | -1.289 | 0.203 |
|  | R |  | -0.523 | 0.603 |  | 0.794 | 0.431 |  | -0.804 | 0.425 |
| Lingual | L |  | -1.665 | 0.102 |  | 1.227 | 0.225 |  | -0.743 | 0.461 |
|  | R |  | -0.369 | 0.713 |  | 0.925 | 0.359 |  | -0.116 | 0.908 |
| Medial orbito-frontal | L |  | **-2.179** | **0.034** |  | -1.100 | 0.276 |  | **-2.349** | **0.023** |
|  | R |  | -1.961 | 0.055 |  | 0.117 | 0.908 |  | -1.818 | 0.075 |
| Middle temporal | L |  | **-2.584** | **0.013** |  | 0.519 | 0.606 |  | **-2.091** | **0.041** |
|  | R |  | **-2.264** | **0.028** |  | 1.295 | 0.201 |  | -1.935 | 0.058 |
| Parahippocampal | L |  | -1.214 | 0.230 |  | **-2.987** | **0.004** |  | **-2.294** | **0.026** |
|  | R |  | **-2.486** | **0.016** |  | **-2.017** | **0.049** |  | **-3.722** | **<0.001** |
| Paracentral | L |  | -0.178 | 0.860 |  | 0.290 | 0.773 |  | -0.461 | 0.646 |
|  | R |  | 0.737 | 0.465 |  | 0.317 | 0.753 |  | 0.173 | 0.863 |
| Pars opercularis | L |  | -1.989 | 0.052 |  | **-2.260** | **0.028** |  | **-3.250** | **0.002** |
|  | R |  | -1.593 | 0.117 |  | -0.497 | 0.621 |  | -2.081 | 0.042 |
| Pars orbitalis | L |  | -0.271 | 0.788 |  | 0.361 | 0.720 |  | -0.212 | 0.833 |
|  | R |  | 0.626 | 0.534 |  | 1.581 | 0.120 |  | 0.698 | 0.488 |
| Pars triangularis | L |  | 0.258 | 0.798 |  | 0.293 | 0.771 |  | 0.298 | 0.767 |
|  | R |  | -1.571 | 0.122 |  | 0.506 | 0.615 |  | -1.270 | 0.210 |
| Pericalcarine | L |  | -1.906 | 0.062 |  | 1.094 | 0.279 |  | -1.151 | 0.255 |
|  | R |  | -1.209 | 0.232 |  | 0.655 | 0.515 |  | -0.842 | 0.403 |
| Postcentral | L |  | 0.082 | 0.935 |  | 0.084 | 0.933 |  | -0.178 | 0.860 |
|  | R |  | 0.740 | 0.463 |  | -0.547 | 0.587 |  | 0.322 | 0.749 |
| Posterior cingulate | L |  | 0.384 | 0.703 |  | -0.848 | 0.400 |  | -0.179 | 0.859 |
|  | R |  | 0.466 | 0.643 |  | -0.612 | 0.543 |  | -0.072 | 0.943 |
| Precentral | L |  | -0.640 | 0.525 |  | -0.372 | 0.711 |  | -0.646 | 0.521 |
|  | R |  | -1.345 | 0.184 |  | 0.561 | 0.577 |  | -1.055 | 0.297 |
| Precuneus | L |  | 0.219 | 0.828 |  | -0.062 | 0.951 |  | -0.101 | 0.920 |
|  | R |  | 0.625 | 0.535 |  | 0.721 | 0.474 |  | 0.617 | 0.540 |
| Rostral anterior cingulate | L |  | **-2.435** | **0.018** |  | -1.076 | 0.287 |  | **-2.843** | **0.006** |
|  | R |  | -1.857 | 0.069 |  | -1.089 | 0.281 |  | **-3.297** | **0.002** |
| Rostral middle frontal | L |  | -1.410 | 0.165 |  | 0.558 | 0.579 |  | -1.010 | 0.317 |
|  | R |  | -1.030 | 0.308 |  | 1.425 | 0.160 |  | -0.649 | 0.519 |
| Superior frontal | L |  | -1.163 | 0.250 |  | -0.370 | 0.713 |  | -1.278 | 0.207 |
|  | R |  | -1.606 | 0.114 |  | 0.719 | 0.475 |  | -1.457 | 0.151 |
| Superior parietal | L |  | -0.341 | 0.735 |  | 1.356 | 0.181 |  | 0.381 | 0.705 |
|  | R |  | -0.377 | 0.708 |  | 1.303 | 0.198 |  | 0.129 | 0.898 |
| Superior temporal | L |  | **-2.244** | **0.029** |  | **-2.271** | **0.027** |  | **-2.930** | **0.005** |
|  | R |  | **-3.117** | **0.003** |  | -0.572 | 0.570 |  | **-2.828** | **0.007** |
| Supramarginal | L |  | 1.248 | 0.218 |  | -0.820 | 0.416 |  | 0.812 | 0.421 |
|  | R |  | 0.361 | 0.720 |  | -1.074 | 0.288 |  | -0.448 | 0.656 |
| Frontal pole | L |  | -0.586 | 0.560 |  | 1.181 | 0.243 |  | 0.845 | 0.402 |
|  | R |  | -0.437 | 0.664 |  | 0.992 | 0.326 |  | 0.754 | 0.454 |
| Temporal pole | L |  | **-4.598** | **0.001** |  | 1.140 | 0.259 |  | -1.613 | 0.113 |
|  | R |  | **-3.019** | **0.004** |  | 1.671 | 0.101 |  | -0.920 | 0.362 |
| Transverse temporal | L |  | -1.081 | 0.285 |  | -1.423 | 0.161 |  | -0.780 | 0.439 |
|  | R |  | -1.667 | 0.101 |  | -0.895 | 0.375 |  | -1.193 | 0.238 |
| Insula | L |  | -1.871 | 0.067 |  | -1.740 | 0.088 |  | **-3.537** | **0.001** |
|  | R |  | **-2.853** | **0.006** |  | **-2.717** | **0.009** |  | **-4.581** | **<0.001** |
| Whole brain | L |  | **-2.050** | **0.045** |  | -0.084 | 0.934 |  | **-2.458** | **0.017** |
|  | R |  | -1.760 | 0.084 |  | 0.464 | 0.645 |  | 0.663 | 0.510 |

**Supplementary Table 1.** Morphometric ROI-based analysis. Effect of the group on the cortical thickness, surface area and cortical volume of temporal structures of the cortex, located in the left (L) and right (R) hemispheres. Data were analyzed by a multivariate linear regression model ($Morph=\beta_{0}+\beta_{1}Center+ \beta_{2}Group+ \beta_{3}Age$) to explore the effect on morphometric values due to the center of acquisition ($Center$), group ($Group$) and age (*Age*). Significative values (*p_value_*<0.05) are indicated in bold.

| **Supplementary Table 2. Structural network-based analysis of surface area**  **and cortical thickness in cortical regions** | | | | | | | | |
| --- | --- | --- | --- | --- | --- | --- | --- | --- |
|  |  |  | Structural network-based analysis | | | | |  |
|  |  |  | Surface  Area [mm^2^] | |  | Cortical  Thickness [mm] | | |
| **Cortical region** | **Side** |  | ***r*_value_** | ***p*_spin_** |  | ***r*_value_** | ***p*_spin_** | |
| Bankssts | L |  | -0.113 | 0.282 |  | -0.022 | 0.554 | |
|  | R |  | -0.076 | 0.216 |  | 0.077 | 0.370 | |
| Caudal anterior cingulate | L |  | 0.116 | 0.249 |  | **-0.282** | **0.038** | |
|  | R |  | 0.065 | 0.435 |  | **-0.227** | **0.037** | |
| Caudal middle frontal | L |  | -0.075 | 0.378 |  | 0.119 | 0.155 | |
|  | R |  | -0.010 | 0.378 |  | -0.002 | 0.417 | |
| Cuneus | L |  | 0.026 | 0.437 |  | 0.149 | 0.133 | |
|  | R |  | 0.006 | 0.553 |  | 0.160 | 0.189 | |
| Entorhinal | L |  | **-0.282** | **0.073** |  | -0.182 | 0.164 | |
|  | R |  | **-0.176** | **0.034** |  | **-0.091** | **0.090** | |
| Fusiform | L |  | **-0.354** | **0.025** |  | **0.128** | **0.070** | |
|  | R |  | **-0.126** | **0.096** |  | **0.180** | **0.099** | |
| Inferior parietal | L |  | -0.013 | 0.593 |  | -0.061 | 0.502 | |
|  | R |  | -0.036 | 0.222 |  | 0.146 | 0.205 | |
| Inferior temporal | L |  | **-0.310** | **0.014** |  | 0.070 | 0.130 | |
|  | R |  | **-0.225** | **0.036** |  | **0.201** | **0.076** | |
| Isthmus cingulate | L |  | 0.198 | 0.159 |  | -0.188 | 0.150 | |
|  | R |  | 0.109 | 0.384 |  | 0.099 | 0.321 | |
| Lateral occipital | L |  | **-0.336** | **0.034** |  | 0.098 | 0.151 | |
|  | R |  | -0.073 | 0.217 |  | 0.130 | 0.254 | |
| Lateral orbito-frontal | L |  | -0.132 | 0.345 |  | -0.071 | 0.466 | |
|  | R |  | -0.070 | 0.279 |  | -0.016 | 0.312 | |
| Lingual | L |  | -0.113 | 0.352 |  | -0.074 | 0.437 | |
|  | R |  | -0.108 | 0.228 |  | 0.146 | 0.212 | |
| Medial orbito-frontal | L |  | -0.137 | 0.307 |  | -0.013 | 0.546 | |
|  | R |  | 0.132 | 0.306 |  | -0.107 | 0.223 | |
| Middle temporal | L |  | -0.221 | 0.119 |  | **0.101** | **0.057** | |
|  | R |  | **-0.091** | **0.078** |  | 0.170 | 0.147 | |
| Parahippocampal | L |  | -0.179 | 0.201 |  | 0.001 | 0.279 | |
|  | R |  | -0.014 | 0.308 |  | 0.076 | 0.449 | |
| Paracentral | L |  | **0.471** | **0.004** |  | 0.042 | 0.397 | |
|  | R |  | **0.417** | **0.006** |  | 0.038 | 0.399 | |
| Pars opercularis | L |  | -0.030 | 0.525 |  | -0.085 | 0.374 | |
|  | R |  | 0.121 | 0.357 |  | 0.052 | 0.514 | |
| Pars orbitalis | L |  | -0.009 | 0.625 |  | **-0.252** | **0.061** | |
|  | R |  | -0.090 | 0.122 |  | 0.105 | 0.349 | |
| Pars triangularis | L |  | -0.097 | 0.403 |  | -0.155 | 0.220 | |
|  | R |  | 0.039 | 0.528 |  | 0.093 | 0.364 | |
| Pericalcarine | L |  | -0.009 | 0.506 |  | 0.072 | 0.240 | |
|  | R |  | 0.015 | 0.554 |  | **0.214** | **0.098** | |
| Postcentral | L |  | **0.321** | **0.030** |  | -0.128 | 0.231 | |
|  | R |  | **0.397** | **0.009** |  | 0.011 | 0.518 | |
| Posterior cingulate | L |  | **0.262** | **0.089** |  | -0.086 | 0.336 | |
|  | R |  | **0.411** | **0.013** |  | -0.032 | 0.380 | |
| Precentral | L |  | 0.196 | 0.146 |  | -0.046 | 0.481 | |
|  | R |  | 0.237 | 0.163 |  | 0.003 | 0.575 | |
| Precuneus | L |  | **0.262** | **0.054** |  | -0.114 | 0.280 | |
|  | R |  | 0.223 | 0.158 |  | 0.005 | 0.622 | |
| Rostral anterior cingulate | L |  | 0.146 | 0.220 |  | -0.210 | 0.102 | |
|  | R |  | 0.101 | 0.353 |  | **-0.169** | **0.087** | |
| Rostral middle frontal | L |  | -0.041 | 0.465 |  | 0.103 | 0.146 | |
|  | R |  | 0.059 | 0.477 |  | 0.128 | 0.228 | |
| Superior frontal | L |  | 0.159 | 0.256 |  | -0.153 | 0.192 | |
|  | R |  | 0.225 | 0.242 |  | **-0.181** | **0.085** | |
| Superior parietal | L |  | 0.108 | 0.266 |  | 0.007 | 0.366 | |
|  | R |  | 0.114 | 0.411 |  | 0.196 | 0.148 | |
| Superior temporal | L |  | -0.045 | 0.578 |  | 0.035 | 0.167 | |
|  | R |  | -0.064 | 0.218 |  | 0.176 | 0.113 | |
| Supramarginal | L |  | 0.016 | 0.379 |  | -0.056 | 0.495 | |
|  | R |  | 0.099 | 0.402 |  | 0.077 | 0.387 | |
| Frontal pole | L |  | -0.022 | 0.452 |  | 0.028 | 0.432 | |
|  | R |  | 0.023 | 0.477 |  | -0.012 | 0.504 | |
| Temporal pole | L |  | **-0.366** | **0.018** |  | -0.202 | 0.132 | |
|  | R |  | **-0.283** | **0.015** |  | -0.054 | 0.133 | |
| Transverse temporal | L |  | **0.139** | **0.098** |  | **-0.276** | **0.035** | |
|  | R |  | -0.038 | 0.295 |  | **-0.183** | **0.042** | |
| Insula | L |  | -0.110 | 0.396 |  | -0.106 | 0.434 | |
|  | R |  | **-0.018** | **0.074** |  | 0.188 | 0.129 | |

**Supplementary Table 2.** Structural network-based analysis of surface area (SA) and cortical thickness (CT) in cortical regions. Bold indicates significantly altered measurements (*p_spin_*<0.1).

| **Supplementary Table 3 - Structural network-based analysis**  **of cortical volume in subcortical regions** | | | | | | | | |
| --- | --- | --- | --- | --- | --- | --- | --- | --- |
|  |  |  | Structural network-based analysis | | | | |  |
|  |  |  | Surface  Area [mm^2^] | |  | Cortical  Thickness [mm] | | |
| **Cortical region** | **Side** |  | ***r*_value_** | ***p*_spin_** |  | ***r*_value_** | ***p*_spin_** | |
| Accumbens | L |  | 0.023 | 0.393 |  | -0.177 | 0.170 | |
|  | R |  | 0.002 | 0.593 |  | **-0.234** | **0.012** | |
| Amygdala | L |  | **-0.436** | **0.003** |  | -0.087 | 0.432 | |
|  | R |  | **-0.154** | **0.033** |  | 0.157 | 0.190 | |
| Caudate | L |  | 0.018 | 0.439 |  | -0.039 | 0.562 | |
|  | R |  | 0.210 | 0.272 |  | -0.024 | 0.248 | |
| Hippocampus | L |  | -0.185 | 0.222 |  | -0.056 | 0.511 | |
|  | R |  | -0.069 | 0.204 |  | 0.087 | 0.432 | |
| Pallidum | L |  | 0.001 | 0.385 |  | -0.055 | 0.475 | |
|  | R |  | 0.029 | 0.607 |  | 0.064 | 0.490 | |
| Putamen | L |  | -0.049 | 0.660 |  | -0.117 | 0.401 | |
|  | R |  | **-0.045** | **0.073** |  | 0.196 | 0.101 | |
| Thalamus | L |  | 0.106 | 0.127 |  | -0.104 | 0.358 | |
|  | R |  | 0.108 | 0.338 |  | 0.118 | 0.282 | |

**Supplementary Table 3.** Structural network-based analysis in subcortical regions. Bold indicates significantly altered measurements (*p_spin_*<0.1).

| **Supplementary Table 4. Effect on grade of psychiatric comorbidities of the morphometric variables of cortical structures in PCDH19-related epilepsy patients** | | | | | | | | | | |
| --- | --- | --- | --- | --- | --- | --- | --- | --- | --- | --- |
|  |  |  | **Morphometric variables** | | | | | | | |
|  |  |  | Surface area  (mm^2^) | |  | Cortical thickness (mm) | |  | Cortical volume (mm^3^) | |
| **Cortical region** | **Side** |  | $\beta_{2}$ | ***p*_value_** |  | $\beta_{2}$ | ***p*_value_** |  | $\beta_{2}$ | ***p*_value_** |
| Bankssts | L |  | -0.0018 | 0.320 |  | **-3.2717** | **0.005** |  | -0.0008 | 0.102 |
|  | R |  | -0.0035 | 0.198 |  | -2.7023 | 0.081 |  | -0.0014 | 0.116 |
| Caudal anterior cingulate | L |  | -0.0033 | 0.223 |  | -1.5735 | 0.132 |  | -0.0008 | 0.199 |
|  | R |  | -0.0026 | 0.159 |  | -0.6415 | 0.573 |  | -0.0009 | 0.095 |
| Caudal middle frontal | L |  | -0.0007 | 0.539 |  | -1.5586 | 0.286 |  | -0.0004 | 0.249 |
|  | R |  | 0.0001 | 0.990 |  | -0.1354 | 0.934 |  | -0.0001 | 0.685 |
| Cuneus | L |  | -0.0016 | 0.359 |  | 0.2752 | 0.863 |  | -0.0001 | 0.801 |
|  | R |  | -0.0003 | 0.850 |  | 0.0701 | 0.971 |  | 0.0001 | 0.971 |
| Entorhinal | L |  | **-0.0093** | **0.013** |  | 0.0211 | 0.969 |  | -0.0012 | 0.095 |
|  | R |  | -0.0007 | 0.852 |  | 0.4295 | 0.501 |  | 0.0001 | 0.988 |
| Fusiform | L |  | **-0.0018** | **0.046** |  | -2.7051 | 0.089 |  | **-0.0005** | **0.013** |
|  | R |  | -0.0005 | 0.547 |  | -2.7521 | 0.160 |  | -0.0001 | 0.551 |
| Inferior parietal | L |  | **-0.0015** | **0.003** |  | -1.6350 | 0.250 |  | **-0.0004** | **0.004** |
|  | R |  | -0.0008 | 0.066 |  | -0.7566 | 0.660 |  | -0.0002 | 0.136 |
| Inferior temporal | L |  | **-0.0011** | **0.048** |  | -1.6802 | 0.217 |  | -0.0002 | 0.092 |
|  | R |  | -0.0010 | 0.053 |  | -1.2071 | 0.425 |  | -0.0002 | 0.089 |
| Isthmus cingulate | L |  | -0.0031 | 0.187 |  | -1.6411 | 0.351 |  | -0.0014 | 0.072 |
|  | R |  | -0.0041 | 0.088 |  | -1.9312 | 0.134 |  | **-0.0016** | **0.021** |
| Lateral occipital | L |  | -0.0006 | 0.214 |  | 0.5972 | 0.763 |  | -0.0001 | 0.529 |
|  | R |  | -0.0003 | 0.660 |  | -2.0604 | 0.276 |  | -0.0001 | 0.403 |
| Lateral orbito-frontal | L |  | -0.0009 | 0.252 |  | -1.4775 | 0.254 |  | -0.0005 | 0.099 |
|  | R |  | -0.0012 | 0.110 |  | 1.0872 | 0.553 |  | -0.0005 | 0.112 |
| Lingual | L |  | -0.0003 | 0.647 |  | 0.3748 | 0.819 |  | 0.0001 | 0.961 |
|  | R |  | -0.0001 | 0.865 |  | -2.4632 | 0.297 |  | -0.0001 | 0.819 |
| Medial orbito-frontal | L |  | -0.0019 | 0.156 |  | -0.6011 | 0.713 |  | -0.0006 | 0.185 |
|  | R |  | **-0.0027** | **0.013** |  | -1.8884 | 0.229 |  | **-0.0009** | **0.004** |
| Middle temporal | L |  | -0.0009 | 0.186 |  | -1.7803 | 0.189 |  | -0.0003 | 0.102 |
|  | R |  | -0.0012 | 0.082 |  | -1.2101 | 0.385 |  | -0.0002 | 0.164 |
| Parahippocampal | L |  | -0.0067 | 0.065 |  | -0.2452 | 0.792 |  | -0.0010 | 0.252 |
|  | R |  | -0.0050 | 0.365 |  | -1.3755 | 0.144 |  | -0.0024 | 0.051 |
| Paracentral | L |  | -0.0018 | 0.445 |  | 0.3785 | 0.792 |  | -0.0003 | 0.619 |
|  | R |  | -0.0011 | 0.472 |  | -0.6470 | 0.676 |  | -0.0003 | 0.491 |
| Pars opercularis | L |  | -0.0008 | 0.538 |  | -2.8646 | 0.057 |  | -0.0004 | 0.307 |
|  | R |  | -0.0010 | 0.663 |  | -2.8527 | 0.065 |  | -0.0005 | 0.447 |
| Pars orbitalis | L |  | -0.0042 | 0.127 |  | -0.8569 | 0.335 |  | -0.0007 | 0.145 |
|  | R |  | -0.0066 | 0.060 |  | -1.1918 | 0.425 |  | -0.0011 | 0.101 |
| Pars triangularis | L |  | **-0.0040** | **0.005** |  | -1.9856 | 0.247 |  | **-0.0010** | **0.017** |
|  | R |  | -0.0017 | 0.337 |  | 0.0607 | 0.976 |  | -0.0001 | 0.751 |
| Pericalcarine | L |  | -0.0003 | 0.777 |  | 0.2673 | 0.887 |  | 0.0001 | 0.782 |
|  | R |  | -0.0001 | 0.891 |  | -1.0452 | 0.530 |  | -0.0001 | 0.821 |
| Postcentral | L |  | -0.0015 | 0.128 |  | -2.1044 | 0.321 |  | -0.0004 | 0.183 |
|  | R |  | -0.0016 | 0.073 |  | -2.1344 | 0.466 |  | -0.0004 | 0.109 |
| Posterior cingulate | L |  | -0.0035 | 0.138 |  | -1.2976 | 0.361 |  | -0.0006 | 0.264 |
|  | R |  | -0.0010 | 0.619 |  | -0.3800 | 0.854 |  | -0.0002 | 0.775 |
| Precentral | L |  | -0.0008 | 0.289 |  | -2.1918 | 0.151 |  | -0.0003 | 0.113 |
|  | R |  | -0.0007 | 0.502 |  | -1.1320 | 0.417 |  | -0.0003 | 0.262 |
| Precuneus | L |  | -0.0012 | 0.054 |  | -1.5397 | 0.388 |  | -0.0003 | 0.062 |
|  | R |  | -0.0009 | 0.161 |  | -1.4463 | 0.433 |  | -0.0003 | 0.132 |
| Rostral anterior cingulate | L |  | **-0.0033** | **0.025** |  | -2.1594 | 0.034 |  | **-0.0010** | **0.005** |
|  | R |  | -0.0042 | 0.117 |  | -1.5573 | 0.068 |  | **-0.0023** | **0.001** |
| Rostral middle frontal | L |  | **-0.0009** | **0.022** |  | -0.7664 | 0.643 |  | **-0.0003** | **0.041** |
|  | R |  | -0.0004 | 0.241 |  | 1.0403 | 0.581 |  | -0.0001 | 0.304 |
| Superior frontal | L |  | -0.0001 | 0.789 |  | -1.6028 | 0.357 |  | -0.0001 | 0.657 |
|  | R |  | 0.0001 | 0.917 |  | -1.0031 | 0.501 |  | 0.0001 | 0.791 |
| Superior parietal | L |  | -0.0007 | 0.031 |  | -0.9358 | 0.582 |  | -0.0002 | 0.051 |
|  | R |  | -0.0007 | 0.083 |  | 0.6290 | 0.747 |  | -0.0001 | 0.250 |
| Superior temporal | L |  | -0.0005 | 0.655 |  | -3.9567 | 0.007 |  | -0.0003 | 0.191 |
|  | R |  | -0.0008 | 0.567 |  | -3.8243 | 0.009 |  | -0.0005 | 0.083 |
| Supramarginal | L |  | **-0.0012** | **0.026** |  | -1.1173 | 0.455 |  | -0.0003 | 0.061 |
|  | R |  | -0.0012 | 0.057 |  | -2.8847 | 0.159 |  | **-0.0003** | **0.048** |
| Frontal pole | L |  | -0.0138 | 0.148 |  | -0.0497 | 0.952 |  | -0.0009 | 0.516 |
|  | R |  | -0.0070 | 0.328 |  | 0.7525 | 0.338 |  | 0.0007 | 0.444 |
| Temporal pole | L |  | -0.0053 | 0.274 |  | -0.0646 | 0.944 |  | -0.0007 | 0.338 |
|  | R |  | -0.0043 | 0.359 |  | -0.7581 | 0.286 |  | -0.0008 | 0.224 |
| Transverse temporal | L |  | 0.0025 | 0.633 |  | -1.9566 | 0.014 |  | -0.0014 | 0.228 |
|  | R |  | 0.0134 | 0.102 |  | **-2.4586** | **0.001** |  | -0.0026 | 0.152 |
| Insula | L |  | -0.0018 | 0.144 |  | -1.0201 | 0.312 |  | **-0.0007** | **0.033** |
|  | R |  | -0.0022 | 0.141 |  | -1.2550 | 0.329 |  | -0.0006 | 0.091 |

**Supplementary Table 4**. Effect on grade of psychiatric comorbidities of the cortical thickness, surface area and cortical volume of structures in *PCDH19*-related epilepsy patients. Data were analyzed by a multivariate linear regression model ($Grade=\beta_{0}+\beta_{1}Center+ \beta_{2}Morph+ \beta_{3}Age$) to explore the effect on the clinical variables of patients due to the center of acquisition ($Center$), group ($Group$) and age (*Age*). Bold indicates significantly altered measures (*p*<0.05).

| **Supplementary Table 5. Effect on the habitual seizures on the morphometric variables of cortical structures in PCDH19-related epilepsy patients** | | | | | | | | | | |
| --- | --- | --- | --- | --- | --- | --- | --- | --- | --- | --- |
|  |  |  | **Morphometric variables** | | | | | | | |
|  |  |  | Surface area  (mm^2^) | |  | Cortical thickness (mm) | |  | Cortical volume  (mm^3^) | |
| **Cortical region** | **Side** |  | $\beta_{2}$ | ***p*_value_** |  | $\beta_{2}$ | ***p*_value_** |  | $\beta_{2}$ | ***p*_value_** |
| Bankssts | L |  | -0.0002 | 0.837 |  | 0.2152 | 0.722 |  | -0.00001 | 0.977 |
|  | R |  | 0.0006 | 0.655 |  | -0.3407 | 0.646 |  | 0.00022 | 0.611 |
| Caudal anterior cingulate | L |  | 0.0017 | 0.163 |  | **1.1745** | **0.008** |  | **0.00057** | **0.029** |
|  | R |  | -0.0010 | 0.226 |  | 0.6070 | 0.236 |  | -0.00011 | 0.672 |
| Caudal middle frontal | L |  | -0.0006 | 0.211 |  | 0.3084 | 0.650 |  | -0.00008 | 0.602 |
|  | R |  | 0.0002 | 0.661 |  | 0.0985 | 0.895 |  | 0.00010 | 0.438 |
| Cuneus | L |  | -0.0004 | 0.605 |  | -0.1242 | 0.865 |  | -0.00011 | 0.660 |
|  | R |  | -0.0002 | 0.752 |  | 0.1148 | 0.897 |  | -0.00002 | 0.912 |
| Entorhinal | L |  | 0.0003 | 0.880 |  | -0.2821 | 0.248 |  | -0.00024 | 0.491 |
|  | R |  | 0.0007 | 0.685 |  | 0.2232 | 0.445 |  | 0.00037 | 0.173 |
| Fusiform | L |  | -0.0003 | 0.463 |  | -0.0923 | 0.904 |  | -0.00007 | 0.539 |
|  | R |  | -0.0001 | 0.892 |  | 1.4904 | 0.092 |  | 0.00004 | 0.702 |
| Inferior parietal | L |  | -0.0001 | 0.642 |  | 0.0151 | 0.982 |  | -0.00002 | 0.761 |
|  | R |  | -0.0002 | 0.390 |  | -0.2444 | 0.757 |  | -0.00004 | 0.462 |
| Inferior temporal | L |  | 0.0001 | 0.780 |  | 0.1545 | 0.809 |  | 0.00002 | 0.773 |
|  | R |  | -0.0001 | 0.689 |  | 0.4465 | 0.521 |  | -0.00001 | 0.895 |
| Isthmus cingulate | L |  | -0.0014 | 0.200 |  | 0.0504 | 0.951 |  | -0.00030 | 0.404 |
|  | R |  | 0.0000 | 0.971 |  | 0.3222 | 0.597 |  | 0.00020 | 0.554 |
| Lateral occipital | L |  | -0.0002 | 0.399 |  | -0.4246 | 0.639 |  | -0.00007 | 0.327 |
|  | R |  | -0.0005 | 0.115 |  | -0.0086 | 0.992 |  | -0.00010 | 0.228 |
| Lateral orbito-frontal | L |  | -0.0007 | 0.060 |  | 1.1385 | 0.045 |  | -0.00009 | 0.526 |
|  | R |  | -0.0003 | 0.335 |  | 1.1899 | 0.145 |  | -0.00008 | 0.634 |
| Lingual | L |  | -0.0003 | 0.371 |  | 0.0783 | 0.917 |  | -0.00009 | 0.431 |
|  | R |  | 0.0000 | 0.984 |  | 1.5553 | 0.144 |  | 0.00004 | 0.696 |
| Medial orbito-frontal | L |  | -0.0004 | 0.537 |  | 1.1291 | 0.117 |  | 0.00020 | 0.371 |
|  | R |  | -0.0004 | 0.424 |  | 0.6858 | 0.345 |  | 0.00000 | 0.995 |
| Middle temporal | L |  | -0.0001 | 0.690 |  | 0.0097 | 0.988 |  | -0.00005 | 0.529 |
|  | R |  | -0.0003 | 0.378 |  | 0.2077 | 0.747 |  | -0.00004 | 0.620 |
| Parahippocampal | L |  | 0.0012 | 0.512 |  | 0.0085 | 0.984 |  | 0.00032 | 0.417 |
|  | R |  | -0.0024 | 0.346 |  | 0.5382 | 0.216 |  | 0.00053 | 0.367 |
| Paracentral | L |  | 0.0003 | 0.755 |  | -0.1675 | 0.799 |  | 0.00006 | 0.825 |
|  | R |  | -0.0003 | 0.702 |  | -0.2968 | 0.676 |  | -0.00005 | 0.806 |
| Pars opercularis | L |  | 0.0000 | 0.942 |  | 0.5327 | 0.463 |  | 0.00008 | 0.639 |
|  | R |  | 0.0010 | 0.324 |  | -0.1159 | 0.877 |  | 0.00020 | 0.452 |
| Pars orbitalis | L |  | -0.0001 | 0.931 |  | 0.3816 | 0.349 |  | 0.00009 | 0.696 |
|  | R |  | -0.0007 | 0.687 |  | -0.1095 | 0.874 |  | -0.00019 | 0.561 |
| Pars triangularis | L |  | -0.0001 | 0.939 |  | 0.5474 | 0.492 |  | 0.00008 | 0.714 |
|  | R |  | -0.0009 | 0.281 |  | 0.9086 | 0.312 |  | -0.00007 | 0.693 |
| Pericalcarine | L |  | -0.0005 | 0.243 |  | -0.9748 | 0.246 |  | -0.00034 | 0.145 |
|  | R |  | -0.0002 | 0.674 |  | -0.3111 | 0.685 |  | -0.00012 | 0.640 |
| Postcentral | L |  | 0.0000 | 0.940 |  | 0.5921 | 0.546 |  | 0.00008 | 0.550 |
|  | R |  | -0.0008 | 0.044 |  | 0.8395 | 0.532 |  | -0.00013 | 0.330 |
| Posterior cingulate | L |  | 0.0004 | 0.740 |  | **1.5761** | **0.008** |  | **0.00059** | **0.015** |
|  | R |  | 0.0001 | 0.888 |  | 1.7210 | 0.054 |  | 0.00032 | 0.230 |
| Precentral | L |  | -0.0005 | 0.151 |  | -0.1706 | 0.813 |  | -0.00010 | 0.214 |
|  | R |  | -0.0002 | 0.623 |  | 0.2184 | 0.735 |  | 0.00004 | 0.700 |
| Precuneus | L |  | 0.0001 | 0.836 |  | -0.1843 | 0.824 |  | 0.00001 | 0.913 |
|  | R |  | -0.0003 | 0.290 |  | 0.2016 | 0.813 |  | -0.00005 | 0.496 |
| Rostral anterior cingulate | L |  | 0.0003 | 0.649 |  | 0.1922 | 0.701 |  | 0.00014 | 0.433 |
|  | R |  | 0.0003 | 0.812 |  | 0.7785 | 0.044 |  | 0.00047 | 0.190 |
| Rostral middle frontal | L |  | -0.0002 | 0.431 |  | -0.0450 | 0.953 |  | -0.00003 | 0.604 |
|  | R |  | -0.0002 | 0.176 |  | -0.0385 | 0.965 |  | -0.00007 | 0.256 |
| Superior frontal | L |  | -0.0003 | 0.229 |  | -0.2871 | 0.722 |  | -0.00005 | 0.355 |
|  | R |  | -0.0001 | 0.761 |  | 0.1422 | 0.836 |  | 0.00001 | 0.913 |
| Superior parietal | L |  | 0.0000 | 0.945 |  | 0.2502 | 0.749 |  | 0.00001 | 0.911 |
|  | R |  | 0.0000 | 0.901 |  | 0.0174 | 0.985 |  | 0.00001 | 0.906 |
| Superior temporal | L |  | 0.0000 | 0.993 |  | 0.5603 | 0.451 |  | 0.00001 | 0.935 |
|  | R |  | 0.0008 | 0.223 |  | 0.1438 | 0.847 |  | 0.00016 | 0.212 |
| Supramarginal | L |  | -0.0001 | 0.615 |  | -0.3945 | 0.566 |  | 0.00025 | 0.678 |
|  | R |  | -0.0003 | 0.347 |  | -0.0618 | 0.949 |  | -0.00004 | 0.627 |
| Frontal pole | L |  | 0.0018 | 0.688 |  | -0.2844 | 0.449 |  | -0.00021 | 0.542 |
|  | R |  | -0.0059 | 0.063 |  | 0.1634 | 0.653 |  | -0.00027 | 0.527 |
| Temporal pole | L |  | -0.0007 | 0.743 |  | -0.0398 | 0.925 |  | 0.00048 | 0.368 |
|  | R |  | -0.0008 | 0.711 |  | -0.3349 | 0.304 |  | -0.00046 | 0.105 |
| Transverse temporal | L |  | 0.0005 | 0.831 |  | 0.4668 | 0.234 |  | 0.00009 | 0.584 |
|  | R |  | 0.0020 | 0.611 |  | 0.4703 | 0.220 |  | **0.00163** | **0.043** |
| Insula | L |  | -0.0009 | 0.115 |  | 0.8369 | 0.060 |  | 0.00000 | 0.295 |
|  | R |  | -0.0004 | 0.528 |  | 0.6837 | 0.243 |  | 0.00009 | 0.611 |

**Supplementary Table 5**. Effect of habitual seizures on the cortical thickness, surface area and cortical volume of structures in *PCDH19*-related epilepsy patients. Data were analyzed by a multivariate linear regression model ($OS=\beta_{0}+\beta_{1}Center+ \beta_{2}Morph+ \beta_{3}Age$) to explore the effect on the clinical variables of patients due to the center of acquisition ($Center$), group ($Group$) and age (*Age*). Bold indicates significantly altered measures (*p*<0.05).

| **Supplementary Table 6. Effect of the number of treatments on the morphometric variables of cortical structures in PCDH19-related epilepsy patients** | | | | | | | | | | |
| --- | --- | --- | --- | --- | --- | --- | --- | --- | --- | --- |
|  |  |  | **Morphometric variables** | | | | | | | |
|  |  |  | Surface area  (mm^2^) | |  | Cortical thickness (mm) | |  | Cortical volume  (mm^3^) | |
| **Cortical region** | **Side** |  | $\beta_{2}$ | ***p*_value_** |  | $\beta_{2}$ | ***p*_value_** |  | $\beta_{2}$ | ***p*_value_** |
| Bankssts | L |  | -0.0006 | 0.787 |  | -0.5888 | 0.713 |  | 4.38E-05 | 9.47E-01 |
|  | R |  | 0.0030 | 0.372 |  | 2.0621 | 0.286 |  | 1.5E-03 | 0.175 |
| Caudal anterior cingulate | L |  | -0.0042 | 0.193 |  | 1.6311 | 0.202 |  | -4.47E-04 | 5.48E-01 |
|  | R |  | -0.0020 | 0.375 |  | 0.7234 | 0.600 |  | -5.2E-04 | 0.435 |
| Caudal middle frontal | L |  | -0.0025 | 0.059 |  | 1.3838 | 0.439 |  | -6.09E-04 | 1.22E-01 |
|  | R |  | -0.0013 | 0.199 |  | 1.3148 | 0.505 |  | -5.2E-04 | 0.116 |
| Cuneus | L |  | 0.0020 | 0.337 |  | 3.0707 | 0.096 |  | 9.07E-04 | 1.40E-01 |
|  | R |  | 0.0017 | 0.296 |  | 2.4301 | 0.292 |  | 6.0E-04 | 0.228 |
| Entorhinal | L |  | -0.0062 | 0.200 |  | **1.4803** | **0.014** |  | 7.82E-04 | 3.84E-01 |
|  | R |  | -0.0004 | 0.930 |  | **1.8152** | **0.010** |  | **1.5E-03** | **0.035** |
| Fusiform | L |  | 0.0001 | 0.958 |  | 1.6025 | 0.422 |  | 1.02E-04 | 7.21E-01 |
|  | R |  | 0.0008 | 0.463 |  | 3.0530 | 0.201 |  | 2.7E-04 | 0.344 |
| Inferior parietal | L |  | 0.0000 | 0.971 |  | 1.4754 | 0.397 |  | 5.94E-05 | 7.50E-01 |
|  | R |  | -0.0001 | 0.797 |  | 2.2267 | 0.277 |  | 6.7E-05 | 0.679 |
| Inferior temporal | L |  | -0.0001 | 0.877 |  | 0.9293 | 0.581 |  | -6.98E-06 | 9.64E-01 |
|  | R |  | -0.0003 | 0.689 |  | 1.7909 | 0.327 |  | -2.4E-05 | 0.880 |
| Isthmus cingulate | L |  | 0.0008 | 0.795 |  | 3.5401 | 0.086 |  | 5.12E-04 | 5.93E-01 |
|  | R |  | 0.0009 | 0.768 |  | 0.4784 | 0.767 |  | 7.7E-05 | 0.933 |
| Lateral occipital | L |  | 0.0004 | 0.568 |  | 5.2426 | 0.017 |  | 2.87E-04 | 1.39E-01 |
|  | R |  | 0.0008 | 0.388 |  | 3.1220 | 0.169 |  | 2.4E-04 | 0.249 |
| Lateral orbito-frontal | L |  | -0.0002 | 0.814 |  | 3.6876 | 0.011 |  | 4.45E-04 | 2.42E-01 |
|  | R |  | 0.0005 | 0.633 |  | 4.0271 | 0.056 |  | 8.2E-04 | 0.040 |
| Lingual | L |  | 0.0010 | 0.267 |  | 3.4878 | 0.064 |  | 4.60E-04 | 9.71E-02 |
|  | R |  | 0.0004 | 0.576 |  | 4.3662 | 0.119 |  | 2.6E-04 | 0.360 |
| Medial orbito-frontal | L |  | 0.0010 | 0.559 |  | 0.5492 | 0.782 |  | 8.40E-04 | 1.36E-01 |
|  | R |  | 0.0000 | 0.987 |  | 0.7328 | 0.706 |  | 1.6E-04 | 0.688 |
| Middle temporal | L |  | -0.0003 | 0.721 |  | 1.9023 | 0.250 |  | 5.21E-05 | 7.98E-01 |
|  | R |  | -0.0001 | 0.870 |  | 2.7257 | 0.096 |  | 1.7E-04 | 0.406 |
| Parahippocampal | L |  | -0.0036 | 0.443 |  | 0.9668 | 0.386 |  | -3.09E-04 | 7.67E-01 |
|  | R |  | -0.0030 | 0.655 |  | 0.7545 | 0.520 |  | 1.0E-04 | 0.948 |
| Paracentral | L |  | 0.0000 | 0.998 |  | 0.6231 | 0.720 |  | -3.09E-04 | 6.79E-01 |
|  | R |  | -0.0002 | 0.934 |  | -0.0574 | 0.976 |  | -2.7E-04 | 0.584 |
| Pars opercularis | L |  | -0.0004 | 0.805 |  | 2.0211 | 0.287 |  | 7.82E-05 | 8.66E-01 |
|  | R |  | 0.0004 | 0.896 |  | 3.5409 | 0.058 |  | 5.7E-04 | 0.430 |
| Pars orbitalis | L |  | 0.0006 | 0.863 |  | 1.0043 | 0.352 |  | 2.97E-04 | 6.16E-01 |
|  | R |  | 0.0065 | 0.136 |  | 1.1178 | 0.539 |  | 1.2E-03 | 0.151 |
| Pars triangularis | L |  | -0.0019 | 0.316 |  | 2.3654 | 0.256 |  | -3.02E-04 | 5.85E-01 |
|  | R |  | 0.0029 | 0.160 |  | 3.1814 | 0.175 |  | 8.7E-04 | 0.067 |
| Pericalcarine | L |  | 0.0005 | 0.666 |  | 2.2868 | 0.306 |  | 4.70E-04 | 4.51E-01 |
|  | R |  | 0.0008 | 0.533 |  | 2.7134 | 0.169 |  | 6.1E-04 | 0.371 |
| Postcentral | L |  | -0.0015 | 0.204 |  | 1.2973 | 0.618 |  | -2.02E-04 | 5.44E-01 |
|  | R |  | -0.0010 | 0.393 |  | 1.3813 | 0.699 |  | -3.5E-04 | 0.313 |
| Posterior cingulate | L |  | -0.0005 | 0.871 |  | 2.5957 | 0.122 |  | 4.59E-04 | 5.10E-01 |
|  | R |  | -0.0004 | 0.852 |  | 2.6760 | 0.277 |  | 3.6E-04 | 0.618 |
| Precentral | L |  | -0.0014 | 0.101 |  | 1.8199 | 0.334 |  | -1.27E-04 | 5.52E-01 |
|  | R |  | -0.0003 | 0.790 |  | 2.0611 | 0.217 |  | 2.8E-04 | 0.324 |
| Precuneus | L |  | 0.0009 | 0.248 |  | 0.6594 | 0.763 |  | 1.98E-04 | 3.47E-01 |
|  | R |  | 0.0010 | 0.188 |  | 2.4396 | 0.270 |  | 2.8E-04 | 0.170 |
| Rostral anterior cingulate | L |  | 0.0000 | 0.986 |  | -0.2799 | 0.833 |  | 7.05E-05 | 8.80E-01 |
|  | R |  | -0.0001 | 0.973 |  | 0.4861 | 0.655 |  | 2.8E-04 | 0.772 |
| Rostral middle frontal | L |  | 0.0003 | 0.624 |  | 1.5879 | 0.425 |  | 1.50E-04 | 3.54E-01 |
|  | R |  | 0.0000 | 0.969 |  | 2.9901 | 0.180 |  | 6.6E-05 | 0.675 |
| Superior frontal | L |  | -0.0007 | 0.254 |  | 0.1855 | 0.931 |  | -2.30E-04 | 1.27E-01 |
|  | R |  | 0.0000 | 0.932 |  | 0.2174 | 0.905 |  | -7.0E-05 | 0.619 |
| Superior parietal | L |  | 0.0002 | 0.616 |  | 2.4344 | 0.229 |  | 9.94E-05 | 4.31E-01 |
|  | R |  | 0.0003 | 0.593 |  | 1.8442 | 0.432 |  | 8.9E-05 | 0.538 |
| Superior temporal | L |  | -0.0011 | 0.383 |  | 2.3508 | 0.225 |  | 4.64E-05 | 8.71E-01 |
|  | R |  | 0.0003 | 0.863 |  | 1.6495 | 0.397 |  | 8.8E-05 | 0.797 |
| Supramarginal | L |  | 0.0003 | 0.665 |  | 1.8905 | 0.293 |  | 1.45E-04 | 4.26E-01 |
|  | R |  | 0.0002 | 0.762 |  | 3.8101 | 0.123 |  | 1.7E-04 | 0.433 |
| Frontal pole | L |  | 0.0034 | 0.777 |  | 0.8845 | 0.372 |  | 1.29E-03 | 4.21E-01 |
|  | R |  | 0.0028 | 0.749 |  | -0.0091 | 0.993 |  | -1.4E-04 | 0.903 |
| Temporal pole | L |  | -0.0010 | 0.864 |  | 0.5561 | 0.616 |  | 5.12E-04 | 5.71E-01 |
|  | R |  | -0.0106 | 0.050 |  | -0.0471 | 0.957 |  | -8.4E-04 | 0.280 |
| Transverse temporal | L |  | 0.0012 | 0.855 |  | 0.3353 | 0.752 |  | 1.47E-03 | 2.95E-01 |
|  | R |  | 0.0163 | 0.101 |  | -0.4406 | 0.671 |  | 2.6E-03 | 0.239 |
| Insula | L |  | -0.0008 | 0.613 |  | 0.5929 | 0.632 |  | -1.01E-04 | 8.14E-01 |
|  | R |  | -0.0006 | 0.756 |  | 0.8799 | 0.576 |  | -1.2E-04 | 0.785 |

**Supplementary Table 6**. Effect of number of treatments on the cortical thickness, surface area and cortical volume of structures in *PCDH19*-related epilepsy patients. Data were analyzed by a multivariate linear regression model ($NT=\beta_{0}+\beta_{1}Center+ \beta_{2}Morph+ \beta_{3}Age$) to explore the effect on the clinical variables of patients due to the center of acquisition ($Center$), group ($Group$) and age (*Age*). Bold indicates significantly altered measures (*p*<0.05).

| **Supplementary Table 7 – Volumetric analysis of hippocampal subfields - Effect of the group on the volume of hippocampal subfields in PCDH19 and controls** | | | | | | | | | | | | | | | | |  |  |  |
| --- | --- | --- | --- | --- | --- | --- | --- | --- | --- | --- | --- | --- | --- | --- | --- | --- | --- | --- | --- |
|  |  |  | Controls | | |  | | PCDH19 | | | |  |  | | | |  |  |  |
| **Region** | **Side** |  | $Mean$ | *STD* | |  | | $Mean$ | | *STD* | |  | $\beta_{2}$ | | ***p*_value_** | |  |  |  |
| Hippocampal tail | **L** |  | 578.29 | | 80.73 | |  | | 543.10 | | 82.23 | | | -1.486 | | 0.143 | | |  |
|  | **R** |  | 583.09 | | 76.97 | |  | | 538.78 | | 74.02 | | | **-2.021** | | **0.048** | | |  |
| Subiculum body | **L** |  | 237.96 | | 33.16 | |  | | 226.71 | | 44.80 | | | -0.995 | | 0.324 | | |  |
|  | **R** |  | 226.95 | | 30.34 | |  | | 211.58 | | 35.51 | | | -1.658 | | 0.103 | | |  |
| CA1 body | **L** |  | 122.01 | | 19.90 | |  | | 112.15 | | 21.91 | | | -1.656 | | 0.103 | | |  |
|  | **R** |  | 134.35 | | 22.07 | |  | | 124.06 | | 16.95 | | | -1.726 | | 0.089 | | |  |
| CA1 head | **L** |  | 503.41 | | 64.89 | |  | | 502.35 | | 57.96 | | | -0.007 | | 0.995 | | |  |
|  | **R** |  | 534.12 | | 71.88 | |  | | 531.68 | | 65.73 | | | -0.061 | | 0.951 | | |  |
| Pre-subiculum head | **L** |  | 135.48 | | 17.08 | |  | | 134.50 | | 13.06 | | | -0.243 | | 0.809 | | |  |
|  | **R** |  | 134.58 | | 16.78 | |  | | 133.50 | | 14.44 | | | -0.201 | | 0.841 | | |  |
| Pre-subiculum body | **L** |  | 170.26 | | 24.92 | |  | | 166.34 | | 32.26 | | | -0.427 | | 0.671 | | |  |
|  | **R** |  | 156.40 | | 22.18 | |  | | 148.50 | | 23.38 | | | -1.177 | | 0.244 | | |  |
| Parasubiculum | **L** |  | 63.90 | | 8.79 | |  | | 63.86 | | 7.62 | | | -0.050 | | 0.961 | | |  |
|  | **R** |  | 64.78 | | 7.89 | |  | | 62.77 | | 9.76 | | | -0.793 | | 0.431 | | |  |
| Hippocampal fissure | **L** |  | 132.59 | | 21.63 | |  | | 114.09 | | 20.26 | | | **-3.087** | | **0.003** | | |  |
|  | **R** |  | 134.22 | | 21.10 | |  | | 118.68 | | 22.43 | | | **-2.530** | | **0.014** | | |  |
| Molecular layer head | **L** |  | 322.68 | | 38.22 | |  | | 316.87 | | 36.04 | | | -0.495 | | 0.623 | | |  |
|  | **R** |  | 336.60 | | 45.01 | |  | | 332.73 | | 41.38 | | | -0.252 | | 0.801 | | |  |
| Molecular layer body | **L** |  | 225.81 | | 29.79 | |  | | 209.30 | | 30.07 | | | -1.938 | | 0.058 | | |  |
|  | **R** |  | 232.22 | | 30.05 | |  | | 212.81 | | 21.58 | | | **-2.472** | | **0.016** | | |  |
| GC ML DG head | **L** |  | 150.04 | | 19.84 | |  | | 143.51 | | 20.07 | | | -1.104 | | 0.274 | | |  |
|  | **R** |  | 160.23 | | 25.07 | |  | | 151.20 | | 20.98 | | | -1.288 | | 0.203 | | |  |
| GC ML DG body | **L** |  | 132.31 | | 18.24 | |  | | 125.06 | | 20.80 | | | -1.283 | | 0.205 | | |  |
|  | **R** |  | 134.83 | | 17.12 | |  | | 122.17 | | 13.09 | | | **-2.802** | | **0.007** | | |  |
| Fimbria | **L** |  | 74.88 | | 19.00 | |  | | 88.61 | | 23.42 | | | **2.325** | | **0.024** | | |  |
|  | **R** |  | 73.29 | | 16.67 | |  | | 73.67 | | 12.11 | | | 0.018 | | 0.985 | | |  |
| HATA | **L** |  | 57.21 | | 9.09 | |  | | 60.02 | | 12.48 | | | 0.940 | | 0.352 | | |  |
|  | **R** |  | 61.34 | | 9.85 | |  | | 56.53 | | 12.12 | | | -1.664 | | 0.101 | | |  |
| CA3 head | **L** |  | 85.62 | | 16.98 | |  | | 71.51 | | 11**.72** | | | **-3.230** | | **0.002** | | |  |
|  | **R** |  | 130.14 | | 25.13 | |  | | 118.12 | | 20.15 | | | -1.7552 | | 0.084 | | |  |
| CA3 body | **L** |  | 119.08 | | 17.04 | |  | | 110.21 | | 17.11 | | | -1.794 | | 0.078 | | |  |
|  | **R** |  | 95.62 | | 17.13 | |  | | 82.76 | | 10.92 | | | **-2.936** | | **0.004** | | |  |
| CA4 head | **L** |  | 126.13 | | 15.91 | |  | | 118.53 | | 17.04 | | | -1.604 | | 0.115 | | |  |
|  | **R** |  | 134.25 | | 20.28 | |  | | 125.19 | | 18.35 | | | -1.580 | | 0.119 | | |  |
| CA4 body | **L** |  | 117.32 | | 17.50 | |  | | 108.56 | | 16.38 | | | -1.764 | | 0.083 | | |  |
|  | **R** |  | 120.22 | | 16.67 | |  | | 108.79 | | 12.12 | | | **-2.618** | | **0.011** | | |  |
| Whole hippocampal head | **L** |  | 1166.16 | | 148.28 | |  | | 1108.24 | | 161.28 | | | -1.286 | | 0.204 | | |  |
| head  WholWe hippocampal body | **R** |  | 1737.66 | | 221.18 | |  | | 1700.33 | | 213.94 | | | -0.548 | | 0.585 | | |  |
|  | **L** |  | 1660.82 | | 193.90 | |  | | 1638.00 | | 188.18 | | | -0.375 | | 0.709 | | |  |
| Whole hippocampal body | **R** |  | 1173.87 | | 145.27 | |  | | 1084.33 | | 118.57 | | | **-2.285** | | **0.026** | | |  |
|  | **L** |  | 3405.28 | | 392.63 | |  | | 3289.33 | | 393.35 | | | -0.984 | | 0.330 | | |  |
| Whole hippocampus | **R** |  | 3494.62 | | 407.85 | |  | | 3323.44 | | 373.46 | | | -1.474 | | 0.146 | | |  |
|  |  |  |  | |  | |  | |  | |  | | |  | |  | | |  |

**Supplementary Table 7.** Volumetric analysis of hippocampal subfields. Effect of the group due to the volume (mm^3^) calculated in the regions of the hippocampal subfields, located in the left (L) and right (R) hemispheres. Data were analyzed by a multivariate linear regression model (${Volume}=\beta_{0}+\beta_{1}Center+ \beta_{2}Group+ \beta_{3} Age+ \beta_{4} eICV$) to explore the effect on the volume of the hippocampal subfields of the ($Group$), with the center of acquisition ($Center$) and age ($Age$) considered as covariates. Bold indicates significantly altered measures (*p*<0.05).

| **Supplementary Table 8 – Volumetric analysis of amygdala subfields - Effect of the group on the volume of amygdala subfields in PCDH19 and controls** | | | | | | | | | | | | | |  | |  |
| --- | --- | --- | --- | --- | --- | --- | --- | --- | --- | --- | --- | --- | --- | --- | --- | --- |
|  |  |  | Controls | |  | PCDH19 | | |  | |  | | |  | |  |
| **Region** | **Side** |  | $Mean$ | *STD* |  | $Mean$ | *STD* | |  | | $\beta_{2}$ | ***p*_value_** | |  | |  |
| Lateral nucleus | **L** |  | 603.08 | 70.59 |  | 595.60 | | 65.96 | | -0.359 | | | 0.721 | |  | |
|  | **R** |  | 618.98 | 77.02 |  | 611.83 | | 64.61 | | -0.330 | | | 0.743 | |  | |
| Basal nucleus | **L** |  | 417.49 | 45.21 |  | 415.91 | | 46.89 | | -0.097 | | | 0.923 | |  | |
|  | **R** |  | 437.74 | 51.47 |  | 426.45 | | 51.69 | | -0.744 | | | 0.460 | |  | |
| Accessory Basal nucleus | **L** |  | 252.12 | 30.18 |  | 246.61 | | 33.56 | | -0.595 | | | 0.554 | |  | |
|  | **R** |  | 266.62 | 33.68 |  | 261.53 | | 37.55 | | -0.467 | | | 0.642 | |  | |
| Anterior amygdaloid area | **L** |  | 52.22 | 6.03 |  | 50.50 | | 4.54 | | -1.098 | | | 0.277 | |  | |
|  | **R** |  | 54.74 | 7.08 |  | 54.21 | | 7.31 | | -0.215 | | | 0.830 | |  | |
| Central nucleus | **L** |  | 42.65 | 8.88 |  | 38.65 | | 6.64 | | -1.675 | | | 0.100 | |  | |
|  | **R** |  | 45.96 | 9.37 |  | 44.45 | | 8.70 | | -0.540 | | | 0.591 | |  | |
| Medial nucleus | **L** |  | 19.66 | 6.81 |  | 16.70 | | 4.65 | | -1.671 | | | 0.100 | |  | |
|  | **R** |  | 21.66 | 6.71 |  | 20.84 | | 6.36 | | -0.330 | | | 0.742 | |  | |
| Cortical nucleus | **L** |  | 24.68 | 3.80 |  | 22.65 | | 4.21 | | -1.773 | | | 0.082 | |  | |
|  | **R** |  | 26.85 | 4.13 |  | 26.89 | | 4.03 | | 0.163 | | | 0.871 | |  | |
| Cortico-amygdaloid transitio | **L** |  | 172.37 | 19.34 |  | 181.60 | | 27.73 | | 1.431 | | | 0.158 | |  | |
|  | **R** |  | 181.13 | 21.22 |  | 184.41 | | 27.19 | | 0.484 | | | 0.631 | |  | |
| Paralaminar nucleus | **L** |  | 46.86 | 4.73 |  | 47.99 | | 4.92 | | 0.828 | | | 0.412 | |  | |
|  | **R** |  | 48.52 | 5.29 |  | 48.11 | | 5.25 | | -0.255 | | | 0.800 | |  | |
| Whole amygdala | **L** |  | 1631.13 | 179.18 |  | 1616.21 | | 176.13 | | -0.268 | | | 0.790 | |  | |
|  | **R** |  | 1702.20 | 197.00 |  | 1678.72 | | 192.38 | | -0.393 | | | 0.696 | |  | |

**Supplementary Table 8.** Effect of the group due to the volume (mm^3^) calculated in the regions of the amygdala subfields, located in the left (L) and right (R) hemispheres. Data were analyzed by a multivariate linear regression model (${Volume}=\beta_{0}+\beta_{1}Center+ \beta_{2}Group+ \beta_{3} Age+ \beta_{4} eICV$) to explore the effect on the volume of the hippocampal subfields of the ($Group$), with the center of acquisition ($Center$) and age ($Age$) considered as covariates. Bold indicates significantly altered measures (*p*<0.05).

| **Supplementary Table 9 – Effect on clinical variables of the volume of hippocampal sub-structures in PCDH19 patients** | | | | | | | | | | | |  | | |
| --- | --- | --- | --- | --- | --- | --- | --- | --- | --- | --- | --- | --- | --- | --- |
|  | |  |  | **Clinical variables** | | | | | | | |  | |  |
|  | |  |  | Age at  seizure onset | |  | Cognitive  level | |  | Psychiatric disorder | |  |  |  |
| **ROI** | | **Side** |  | $\beta_{2}$ | ***p*_value_** |  | $\beta_{2}$ | ***p*_value_** |  | $\beta_{2}$ | ***p*_value_** |  | |  |
| Hippocampal tail | | **L** |  | 0.0190 | 0.369 |  | -0.0033 | 0.313 |  | -0.0045 | 0.148 |  | |  |
|  | | **R** |  | 0.0303 | 0.207 |  | -0.0030 | 0.432 |  | -0.0038 | 0.293 |  | |  |
| Subiculum body | | **L** |  | -0.0117 | 0.770 |  | -0.0060 | 0.358 |  | **-0.0150** | **0.005** |  | |  |
|  | | **R** |  | 0.0029 | 0.957 |  | -0.0090 | 0.269 |  | -0.0145 | 0.057 |  | |  |
| Subiculum head | | **L** |  | -0.0703 | 0.309 |  | 0.0104 | 0.328 |  | -0.0112 | 0.281 |  | |  |
|  | | **R** |  | -0.1055 | 0.074 |  | 0.0153 | 0.094 |  | -0.0072 | 0.435 |  | |  |
| CA1 body | | **L** |  | 0.0226 | 0.789 |  | -0.0018 | 0.916 |  | 0.0032 | 0.803 |  | |  |
|  | | **R** |  | 0.0930 | 0.364 |  | 0.0026 | 0.870 |  | -0.0178 | 0.247 |  | |  |
| CA1 head | | **L** |  | -0.0168 | 0.570 |  | -0.0001 | 0.980 |  | **-0.0087** | **0.038** |  | |  |
|  | | **R** |  | -0.0315 | 0.217 |  | 0.0022 | 0.575 |  | -0.0067 | 0.072 |  | |  |
| Pre-subiculum head | | **L** |  | -0.0604 | 0.651 |  | 0.0140 | 0.497 |  | -0.0152 | 0.448 |  | |  |
|  | | **R** |  | -0.2220 | 0.048 |  | 0.0318 | 0.068 |  | -0.0092 | 0.609 |  | |  |
| Pre-subiculum body | | **L** |  | -0.0296 | 0.597 |  | -0.005 | 0.552 |  | -0.0135 | 0.096 |  | |  |
|  | | **R** |  | -0.0864 | 0.269 |  | -0.0040 | 0.747 |  | -0.0156 | 0.181 |  | |  |
| Parasubiculum | | **L** |  | -0.3288 | 0.131 |  | 0.0028 | 0.937 |  | -0.0289 | 0.390 |  | |  |
|  | | **R** |  | -0.2662 | 0.133 |  | 0.0203 | 0.469 |  | 0.0008 | 0.976 |  | |  |
| Hippocampal fissure | | **L** |  | **0.2436** | **0.003** |  | -0.0206 | 0.137 |  | -0.0127 | 0.358 |  | |  |
|  | | **R** |  | 0.0426 | 0.611 |  | 0.0146 | 0.250 |  | 0.0056 | 0.655 |  | |  |
| Molecular layer HP head | | **L** |  | -0.0273 | 0.567 |  | -0.0001 | 0.995 |  | **-0.0137** | **0.044** |  | |  |
|  | | **R** |  | -0.0557 | 0.170 |  | 0.0045 | 0.477 |  | -0.0093 | 0.125 |  | |  |
| Molecular layer HP body | | **L** |  | -0.0031 | 0.960 |  | -0.0092 | 0.326 |  | **-0.0171** | **0.049** |  | |  |
|  | | **R** |  | 0.0018 | 0.983 |  | -0.0066 | 0.610 |  | **-0.0272** | **0.021** |  | |  |
| GC ML DG head | | **L** |  | -0.0543 | 0.531 |  | -0.0019 | 0.888 |  | -0.0237 | 0.056 |  | |  |
|  | | **R** |  | -0.1023 | 0.226 |  | 0.0018 | 0.894 |  | -0.0209 | 0.094 |  | |  |
| GC ML DG body | | **L** |  | -0.0389 | 0.650 |  | -0.0059 | 0.657 |  | **-0.0245** | **0.044** |  | |  |
|  | | **R** |  | -0.0281 | 0.845 |  | -0.0059 | 0.790 |  | -0.0330 | 0.112 |  | |  |
| Fimbria | | **L** |  | -0.0986 | 0.168 |  | 0.0194 | 0.077 |  | 0.0057 | 0.608 |  | |  |
|  | | **R** |  | -0.2491 | 0.064 |  | 0.0245 | 0.253 |  | 0.0017 | 0.936 |  | |  |
| HATA | | **L** |  | -0.1473 | 0.282 |  | 0.0116 | 0.587 |  | -0.0158 | 0.447 |  | |  |
|  | | **R** |  | -0.1016 | 0.473 |  | -0.0021 | 0.923 |  | -0.0391 | 0.054 |  | |  |
| CA3 head | | **L** |  | -0.1035 | 0.307 |  | -0.0025 | 0.876 |  | -0.0214 | 0.154 |  | |  |
|  | | **R** |  | -0.1209 | 0.215 |  | -0.0084 | 0.583 |  | **-0.0278** | **0.049** |  | |  |
| CA3 body | | **L** |  | 0.0586 | 0.691 |  | -0.0116 | 0.609 |  | -0.0034 | 0.877 |  | |  |
|  | | **R** |  | 0.1732 | 0.288 |  | -0.0235 | 0.353 |  | -0.0360 | 0.107 |  | |  |
| CA4 head | | **L** |  | -0.0484 | 0.638 |  | -0.0073 | 0.645 |  | **-0.0302** | **0.038** |  | |  |
|  | | **R** |  | -0.1060 | 0.287 |  | 0.0023 | 0.884 |  | -0.0220 | 0.136 |  | |  |
| CA4 body | | **L** |  | -0.0343 | 0.750 |  | -0.0124 | 0.452 |  | **-0.0330** | **0.028** |  | |  |
| Whole hippocampal head |  | **R** |  | -0.0090 | 0.954 |  | -0.0097 | 0.684 |  | -0.0360 | 0.107 |  | |  |
|  |  | **L** |  | -0.0070 | 0.438 |  | -0.0008 | 0.630 |  | -0.0024 | 0.071 |  | |  |
| Whole hippocampal body | | **R** |  | -0.0112 | 0.154 |  | 0.0008 | 0.517 |  | -0.0018 | 0.127 |  | |  |
|  |  | **L** |  | -0.0048 | 0.669 |  | 0.0002 | 0.895 |  | -0.0029 | 0.072 |  | |  |
|  |  | **R** |  | -0.0029 | 0.854 |  | -0.0012 | 0.624 |  | **-0.0045** | **0.048** |  | |  |
| Whole hippocampus | | **L** |  | -0.0016 | 0.718 |  | -0.0002 | 0.739 |  | -0.0012 | 0.055 |  | |  |
|  | | **R** |  | -0.0030 | 0.525 |  | 0.0001 | 0.935 |  | -0.0012 | 0.087 |  | |  |

**Supplementary Table 9.** Effect on the clinical variables of the PCDH19 group due to the volume calculated in the subfields of the left (L) and right (R) hippocampus. Data were analyzed by a multivariate linear regression model (${Clinics}=\beta_{0}+\beta_{1}Center+ \beta_{2}Volume+ \beta_{3} Age$) to explore the effect on the age at seizure onset or the cognitive status or the psychiatric disorder ($Clinics$*,* categorized clinical variables indicated in Supplementary Table 1) of the *Volume*, with the center of acquisition ($Center$) and age ($Age$) considered as covariates. $\beta_{2}$ indicates the regression parameters of the model and represents the slope of the regression lines. Bold indicates significantly altered measures (*p*<0.05).

**Supplementary Table 10 - PCDH19 expression in limbic system structures during mouse embryogenesis**


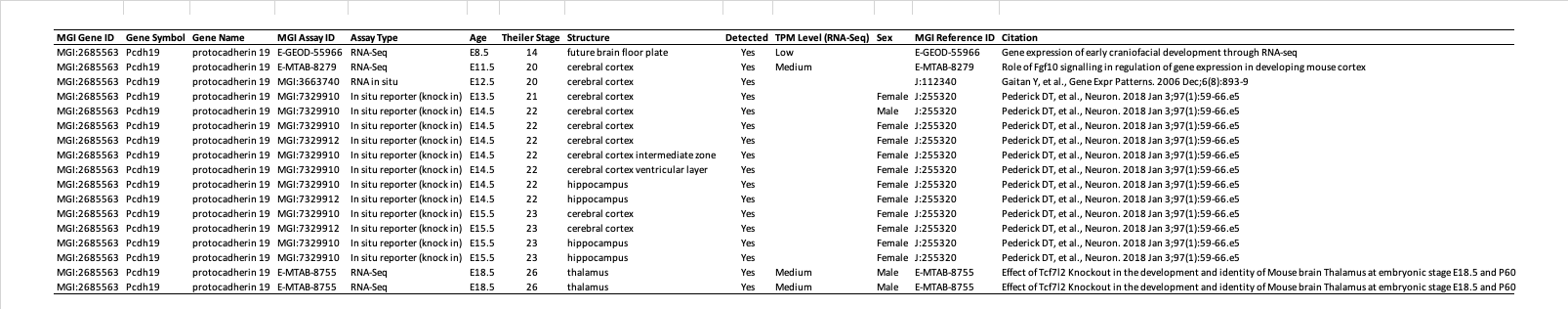


**Supplementary Table 10. PCDH19 expression in limbic system structures during mouse embryogenesis.** Data retrieved from the Mouse Genome Informatics (MGI) database (https://www.informatics.jax.org/) on September 13, 2023. *PCDH19* expression is present in the developing brain from embryonic day (E) 8.5 and has been detected in cerebral cortex (from E11.5 to E15.5), hippocampus (from E14.5 to E15.5), and thalamus (E18.5)

**Supplementary Figures**


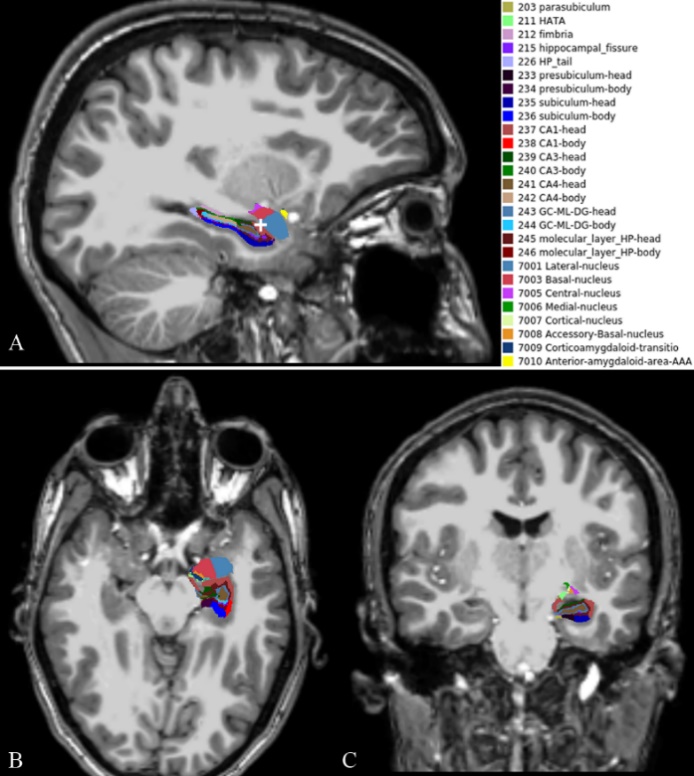


**Supplementary Figure 1.** Hippocampal subfields segmentation. Sagittal (A) plane with the white cross indicating the axial (B) and coronal (C) views of the segmentation of the left hippocampal subfield in patient no. 1.

**
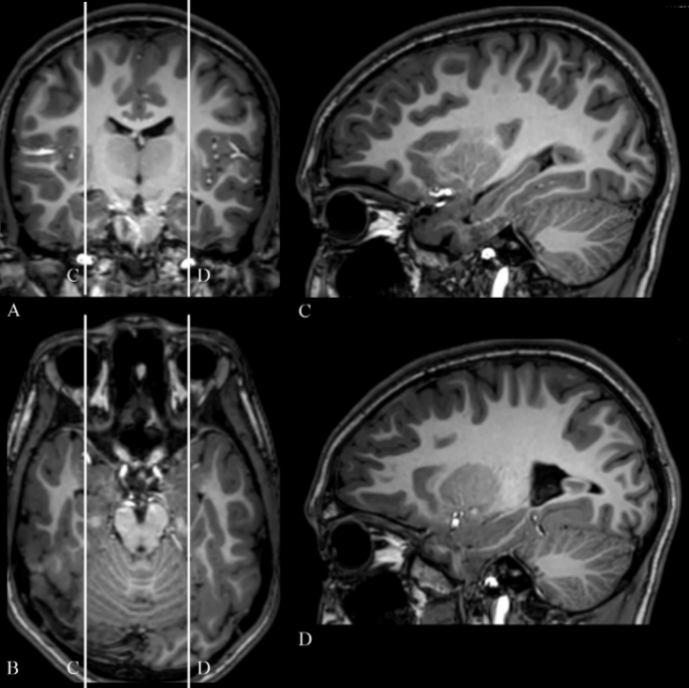
**

**Supplementary Figure 2.** MRI T1-weighted exam of patient no. 10 with incomplete inversion of left hippocampus (D). White lines in coronal (A) and axial (B) MRI views indicates sagittal sections without (C, right hippocampus) and with (D, left hippocampus) incomplete inversion.

**
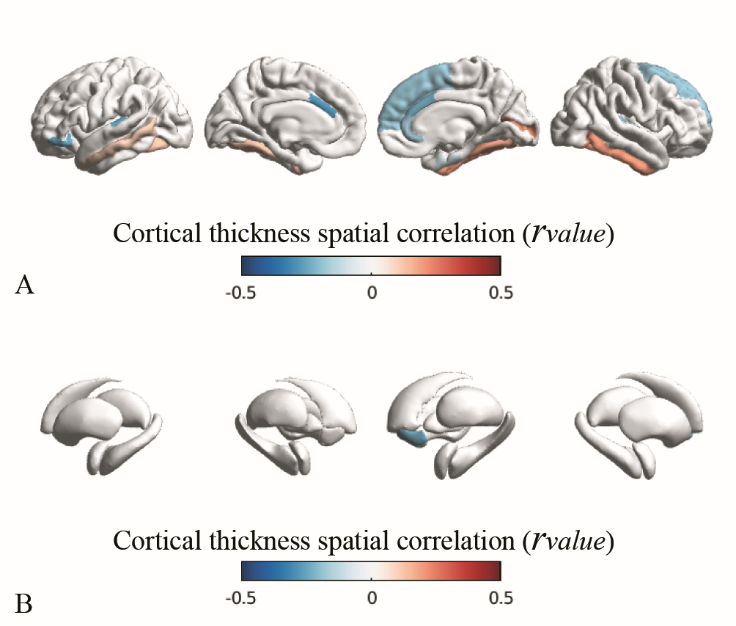
**

**Supplementary Figure 3**. Network-based structural analysis of cortical thickness (CT). Spatial correlations (*r_value_*, blue-red colormap) between CT patterns ($\beta_{2}$) and seed-based cortico-cortical (panel A) and subcortico-cortical (panel B) connectivity profiles. In this example, we used as seed the left entorhinal gyrus. Correlation scores (*r_value_*) are detailed in Supplementary Tables 2 and 3. CT changes ($\beta_{2}$) are indicated in Supplementary Table 1.


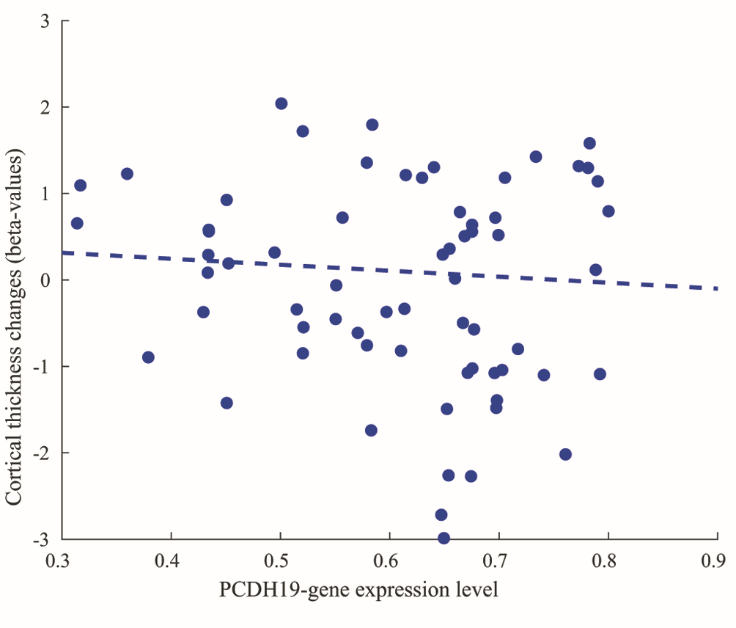


**Supplementary Figure 4.** Correlation between levels of PCDH19 expression and cortical thickness changes ($\beta_{2}$ in Supplementary Table 1) observed in cortical and subcortical regions.


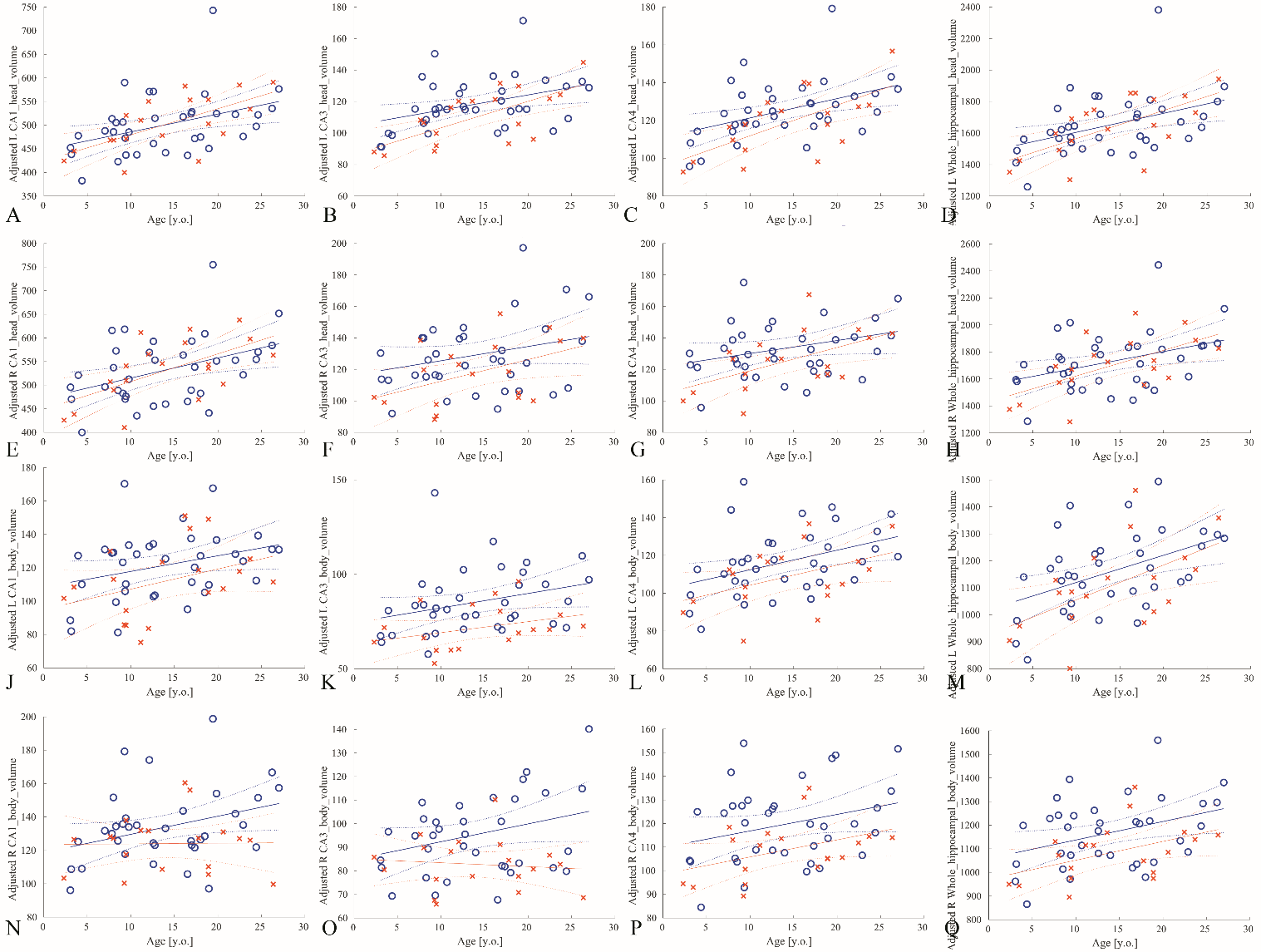


**Supplementary Figure 5.** Age-related cortical patterns of volumes of selected hippocampal subfields, in controls (blue circle) and *PCDH19* mutated patients (red crosses), assessed in the left (L) and right (R) hemispheres. The comparison between the age-related volumetric patterns obtained in the body of CA3 revealed a statistically significantly lower mean value ($\beta_{2}$=-2.936, *p*-value=0.004) and a trend of reduction with age. The body of the whole hippocampus was significantly reduced with age in the right side ($\beta_{0}$=-2.285, *p*-value=0.026). The continuous line represents the fitting line, the dotted line indicated the 95% confidence bounds.


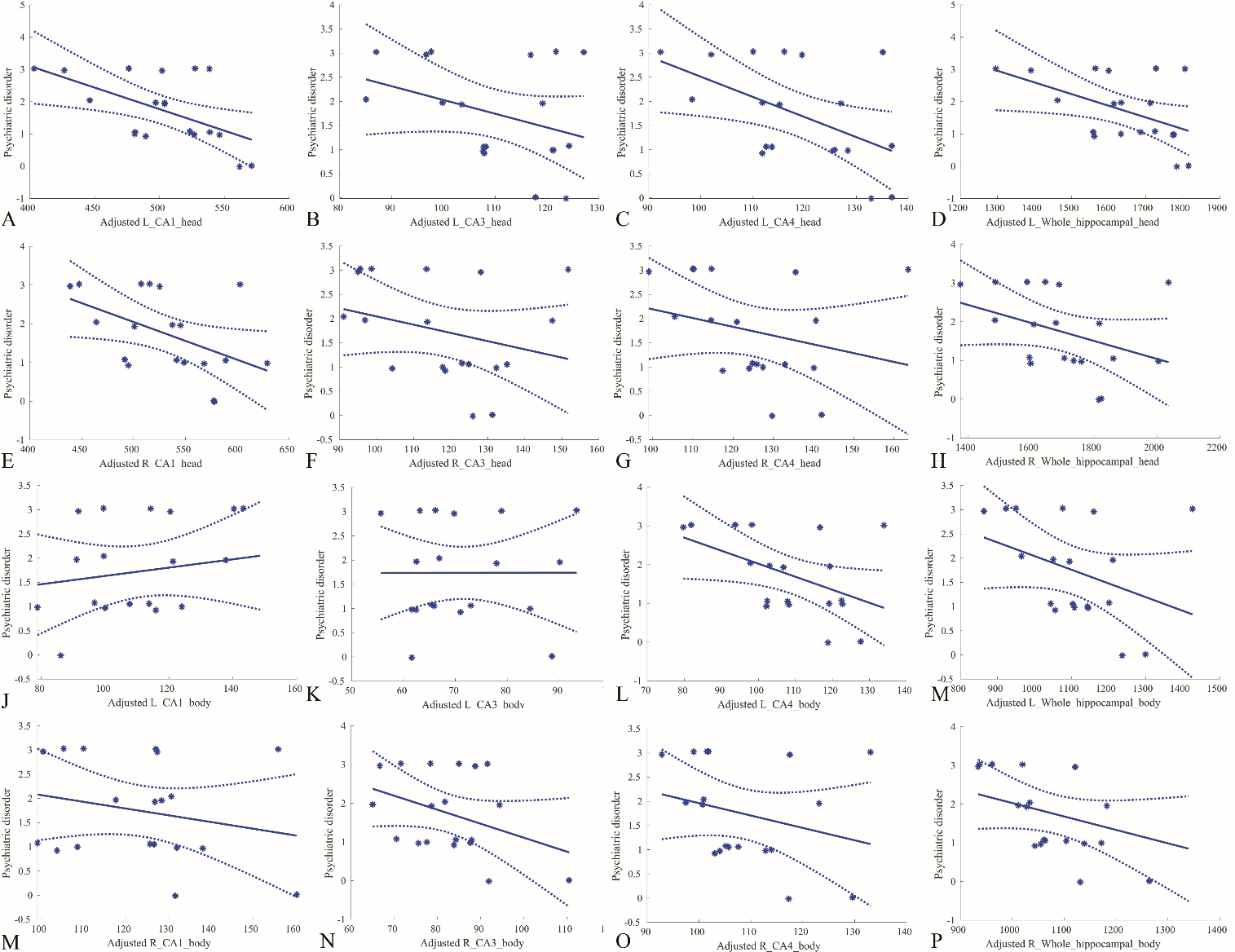


**Supplementary Figure 6.** Significant correlation patterns between the categorized psychiatric disorder and values of volumetric segmentations assessed in a representative subset of the selected regions of interest reported in Table 2. The values, indicated by asterisks, are adjusted according to the linear regression model applied in the statistical analysis. The continuous line represents the fitting line, the dotted line indicated the 95% confidence bounds.

**
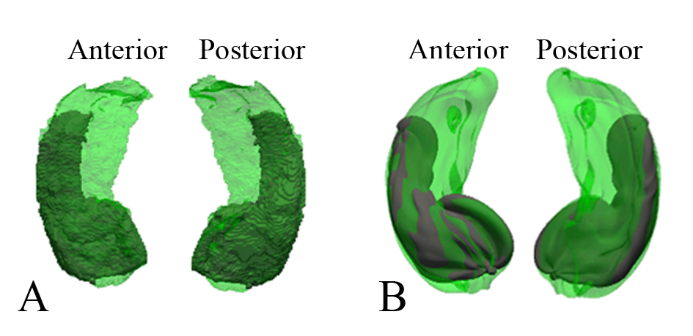
**

**Supplementary Figure 7.** Surface of the left hippocampus of a patient (A, green) after the registration with the surface of the *Cornu Ammonis* (in A, grey). In B, the final shape of the surface of hippocampus (green) and *Cornu Ammonis* (grey) after resampling on the high-resolution mesh (level 6 icosahendreal mesh with 40962 vertices and 81920 faces).

**
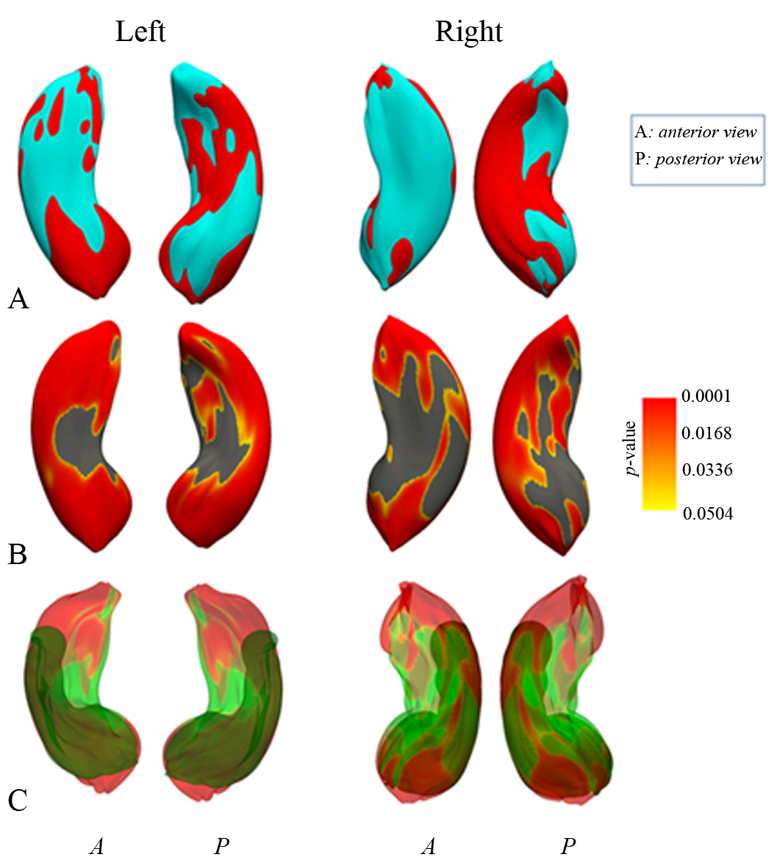
**

**Supplementary Figure 8.** Hippocampal shape analysis. In A, the mean shape of the left and right hippocampi of *PCDH19* *-*mutated patients (red) with in overlay the mean shape of the left and right hippocampi of controls (light blue). In B, the FDRP *p_value_* maps obtained with *p_value_*≤0.05 (yellow-red colormap, with gray for regions not statistically significant, *p_value_*>0.05). In C, the projection of FDRP *p_value_* on mean hippocampus (green) overlaid on the *Cornus Ammonis* (grey).

**
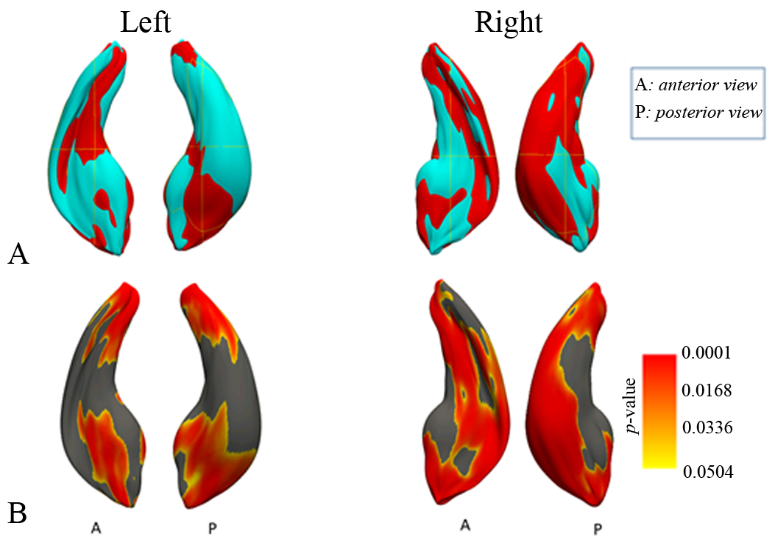
**

**Supplementary Figure 9**. *Cornu Ammonis* (CA) shape analysis. In A, the mean shape of the left and right CA of *PCDH19* patients (red) with in overlay the mean shape of the left and right CA of controls (light blue). In B, the *p*-value maps obtained with *p*≤0.05 (yellow-red colormap, with gray for regions not statistically significant, *p*>0.05).Inizio modulo
